# Epidemiological and antigenic inferences from serological cross-reactivity among arboviruses

**DOI:** 10.1126/scitranslmed.ads8680

**Published:** 2025-11-26

**Authors:** Megan O’Driscoll, Nathanaël Hozé, Noémie Lefrancq, Gabriel Ribeiro dos Santos, Damien Hoinard, Mohammed Ziaur Rahman, Kishor Kumar Paul, Abu Mohd Naser Titu, Mohammad Shafiul Alam, Mohammad Enayet Hossain, Jessica Vanhomwegen, Simon Cauchemez, Emily S. Gurley, Henrik Salje

**Affiliations:** 1 Department of Genetics, University of Cambridge, Cambridge CB23EH, UK; 2 Institute of Global Health, University of Geneva, 1211 Geneva, Switzerland; 3 Mathematical Modelling of Infectious Diseases Unit, Institut Pasteur, INSERM U1332, CNRS UMR2000, Université Paris cité, 75015 Paris, France; 4 UVSQ, INSERM, CESP, Anti-infective Evasion and Pharmacoepidemiology Research Team, Université Paris-Saclay, 78180 Montigny-Le-Bretonneux, France; 5 Department of Biosystems Science and Engineering, ETH Zürich, 4056 Basel, Switzerland; 6 Department of Epidemiology, Johns Hopkins Bloomberg School of Public Health, Baltimore, MD 21205, USA; 7 Environment and Infectious Risks Unit, Institut Pasteur, 75015 Paris, France; 8 International Centre for Diarrhoeal Disease Research, Bangladesh (icddr,b), Dhaka 1213, Bangladesh; 9 School of Population Health, UNSW Sydney, Sydney 2033, Australia; 10 School of Public Health, University of Memphis, Memphis, TN 38152, USA

## Abstract

Multiplex immunoassays can facilitate the parallel measurement of antibody responses against multiple antigenically related pathogens, generating a wealth of high-dimensional data that depict complex antibody-antigen relationships. In this study, we developed a generalizable analytical framework to maximize inferences from multipathogen serological studies. We fit the model to measurements of immunoglobulin antibody binding against 10 arboviral pathogens from a cross-sectional study in northwest Bangladesh with 1453 participants. We used our framework to jointly infer the prevalence of each pathogen by location and age as well as between-pathogen antibody cross-reactivity. Reconstructing immunological profiles, we found evidence of endemic transmission of Japanese encephalitis virus and recent outbreaks of dengue and chikungunya viruses in this district. Our estimates of antibody cross-reactivity were highly correlated with phylogenetic distances inferred from genetic data [correlation coefficient (*r*) = 0.94], demonstrating how antigenic landscapes can be inferred from population-level serological studies. Furthermore, we showed how our framework could be used to identify the presence of antigenically related pathogens that were not directly tested for, representing a potential opportunity for the detection of emerging pathogens. The presented analytical framework offers a tool that can be applied to a growing number of multipathogen studies and will help support the integration of serological testing into disease surveillance platforms.

## Introduction

Traditional infectious disease surveillance methods rely on testing of symptomatic individuals. However, the number of confirmed cases is often a poor indicator of the true underlying burden of infection in a population because of varying rates of subclinical infections, nonspecific symptoms, and heterogeneity in health-seeking behavior (1, 2). Population serological studies, which test for immune markers that are generated in response to an infection, such as antibodies, can provide a direct measure of the underlying burden of infection in a population (3–6). These studies can help to fill the gaps left by traditional disease surveillance methods, allowing an improved understanding of population susceptibility, pathogen transmission dynamics, and rates of pathogen severity (7–9).

Recent advances in high-throughput immunoassay technologies have facilitated the generation of large volumes of immunological data. In particular, multiplex immunoassays, which use color-coded beads to simultaneously measure multiple analytes from a single sample, offer an efficient approach to maximize the information gained from biological samples. In the context of population serological studies, this is leading to a shift in the design of such studies from previously focusing on a single antigen of interest to now being able to simultaneously test for the presence of antibodies against multiple antigens (10–18). By allowing efficient characterization of population immune profiles against multiple pathogens or antigens, such approaches are paving the way for integrated serosurveillance efforts, providing important insights into guiding strategies for the control and prevention of infectious agents (19–21).

Antibody cross-reactivity has long posed a challenge to the interpretation of serological data in regions where antigenically related pathogens cocirculate (22–24). In these contexts, it can be difficult to determine whether antibodies measured against the pathogen of interest were caused by an infection with that specific pathogen or by a related pathogen with similar structural antigenic regions that the antibody can recognize and bind to. Measurements of antibody responses against multiple antigenically related pathogens generate a wealth of high-dimensional data depicting complex antibody-antigen relationships, providing a pathway to characterizing these cross-reactive dynamics (25, 26). For instance, the degree of correlation in antibody responses measured against different antigens at the population level can provide an indication of their antigenic similarity. Analytical methods that quantify and account for these cross-reactive relationships can allow robust characterization of population immune profiles and underlying infection burdens. In addition, cross-reactive relationships provide a potential opportunity to detect emerging pathogens for which it is otherwise not possible to directly test.

Arboviruses, including flaviviruses and alphaviruses, are key examples of diverse viral families that represent ongoing threats to human health. Members of these viral families continue to emerge into the human population, and those that are already established in human transmission cycles continue their geographic expansion, causing increasing burdens to health care systems (27). Monitoring of infection burdens and understanding the antigenic landscape of such viral pathogens are key to global health. In this study, we developed an analytical framework for the analysis of multipathogen serological studies to jointly infer the prevalence of past infections and between-pathogen antibody cross-reactivity. We demonstrate the utility of this framework through application to a cross-sectional serological study of arboviruses in the Chapai Nawabganj district in northwest Bangladesh. Although cases of Japanese encephalitis virus (JEV) are regularly reported in this area and a chikungunya virus (CHIKV) outbreak was documented in recent years, the underlying burden of infection of these and other arboviruses in this district is not well understood (28, 29).

## Results

### Three circulating viruses explained antibody responses against 10 pathogens in a Bangladeshi population

**Fig. 1:**
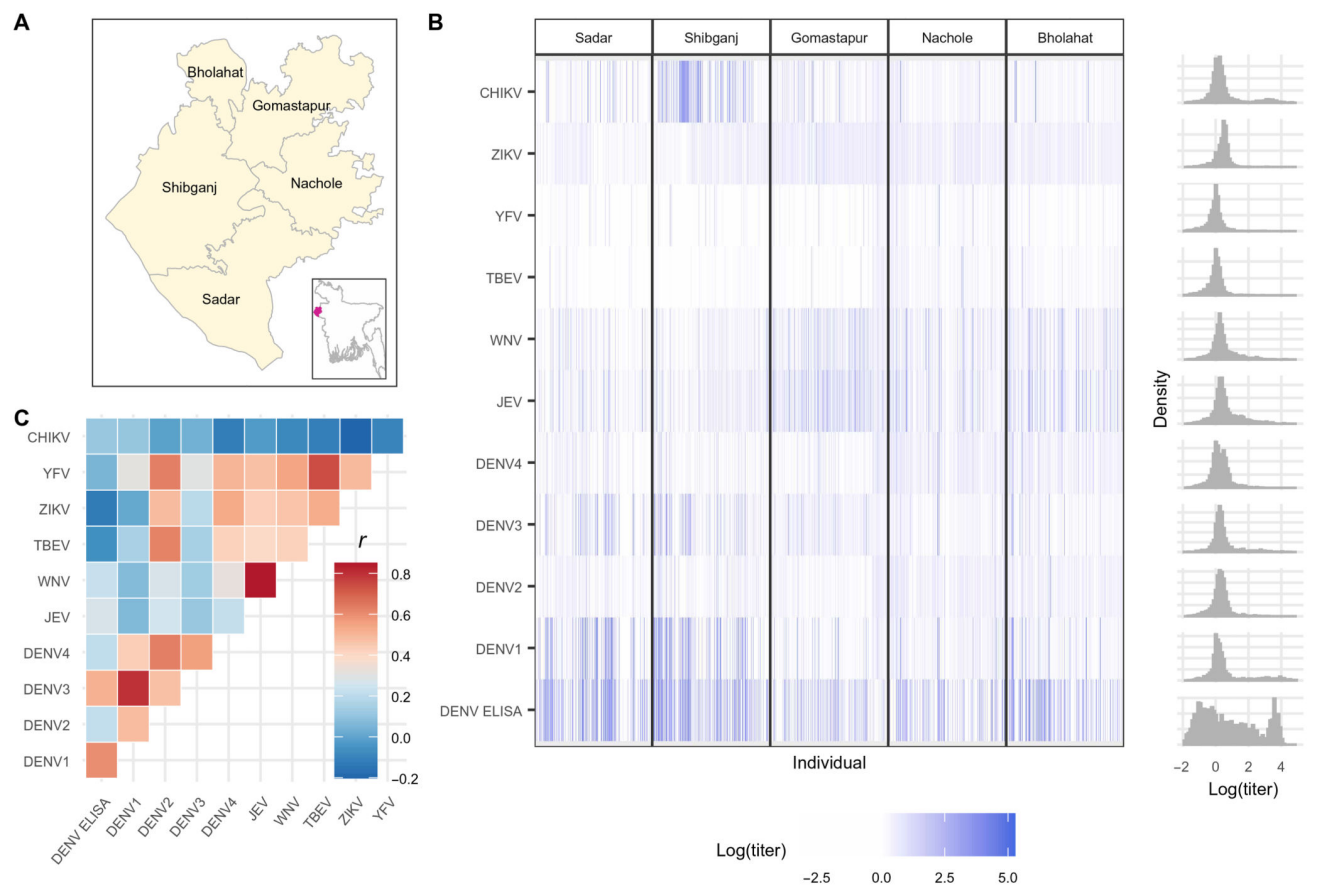
Multipathogen serological study data. (**A**) Map of Chapai Nawabganj district and its five subdistricts. The inset plot shows the location of the Chapai Nawabganj district in Bangladesh, indicated in red. (**B**) Heatmap of the log RFI IgG antibody titer values for multiplex antigens and log titers for the DENV ELISA assay. Column panels show the antibody binding concentrations for each of the five subdistricts. Gray bars on the right show the population distribution of log titer values for each antigen. (**C**) Heatmap of Pearson *r* correlation coefficients of population log antibody titers for each multiplex antigen pair.

We analyzed serological samples from 1453 individuals participating in a cross-sectional study conducted in 2014 in the Chapai Nawabganj district, located in northwest Bangladesh (Fig. 1A). Individuals were enrolled from 39 communities across all five subdistricts with an age range of 2 to 90 years. We used an arbovirus multiplex immunoassay, previously validated using sera from experimentally infected horses (30). Concentrations of binding immunoglobulin G (IgG) antibodies were measured against antigens of 10 pathogens, comprising 9 flaviviruses and 1 alphavirus, using a Luminex platform: dengue virus serotypes 1, 2, 3, and 4 (DENV1 to DENV4); JEV; West Nile virus (WNV); yellow fever virus (YFV); Zika virus (ZIKV); tick-borne encephalitis virus (TBEV); and CHIKV. The domain III of the envelope protein (EDIII) was used as the target antigen for each flavivirus, whereas the E2 protein was used for the alphavirus CHIKV. In addition, samples were tested using a commercial DENV enzyme-linked immunosorbent assay (ELISA) that used the whole E protein. Values of median fluorescence intensity against each antigen from the multiplex immunoassay were divided by individual-level responses to a background control (SNAP-tag) to obtain a relative fluorescence intensity (RFI) measurement. RFIs by antigen and upazila (subdistrict) of residence were determined (Fig. 1B), with respective population distributions of antibody concentrations. Simple Pearson correlation coefficients (*r* values) calculated for RFI titers against each antigen pair revealed high correlations in titers against JEV and WNV (*r* = 0.85), between DENV1 and DENV3 (*r* = 0.80), and between TBEV and YFV (*r* = 0.74) (Fig. 1C and fig. S1). In contrast, we observed low correlation between RFIs measured against CHIKV and all flavivirus antigens, with correlation coefficients ranging from −0.20 to 0.11.

**Fig. 2:**
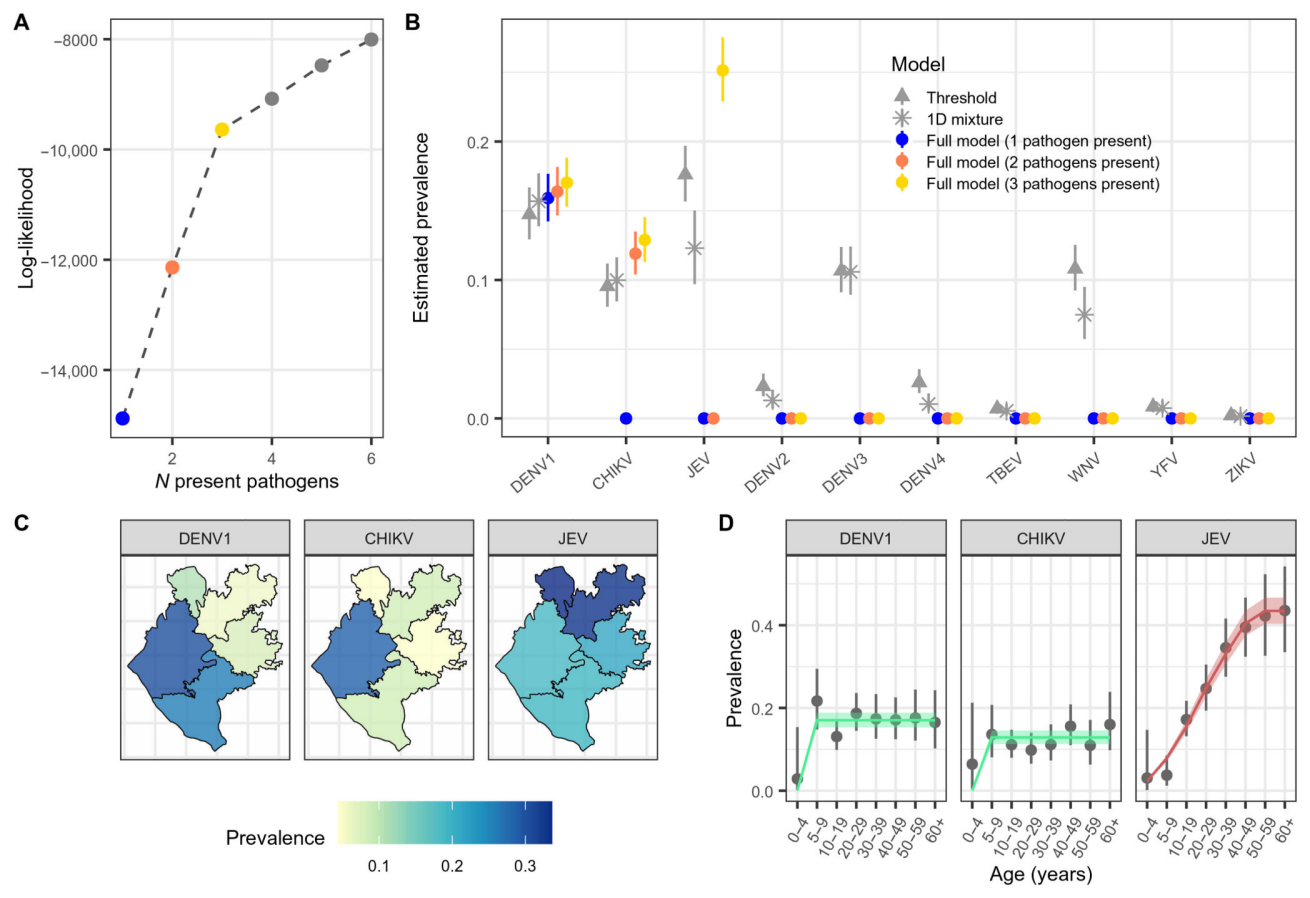
Prevalence of past infections by space and age. (**A**) Increase in log-likelihood estimates from the full model as additional pathogens are assumed to be present. (**B**) A comparison of prevalence estimates across models is shown. Points and lines show the median and 95%Crl estimates of prevalence by pathogen, respectively. The gray points and lines show estimates from a simple mixture model (stars) and threshold method (triangles) fit independently to antibody data for each pathogen. Colored points and lines show estimates from the full model framework, progressing from including one present pathogen (blue) to three present pathogens (yellow). (**C**) Maps of median prevalence estimates by subdistrict for each present pathogen from the final full model. (**D**) Prevalence estimates by age are shown for each present pathogen. Points and lines show the median and 95%Crl estimates of prevalence from the final multivariate mixture model, respectively. The green lines and ribbons show the median and 95%Crl estimates of population prevalence for DENV1 and CHIKV, respectively. The red line and shaded ribbon show the median and 95%Crl estimates from a catalytic model for JEV, respectively, assuming endemic transmission since its estimated introduction time to the region.

We developed a semimechanistic multivariate mixture model to jointly infer pathogen-specific prevalence of past infections, denoted “prevalence” throughout, and between-pathogen antibody cross-reactivity. Mixture models allowed probabilistic inferences of individual infection statuses rather than binary classification, enabling the propagation of uncertainty from the individual level into population-level estimates. Applying our framework to the arbovirus serological data, we conducted a stepwise variable selection process to determine which pathogens were present, i.e., which have transmitted within the study population, and best explain the population antibody titer distributions against all antigens. Fitting to log antibody data from all 11 antigens (multiplex immunoassay and ELISA) in the same framework, we first assumed only a single pathogen to be present such that antibody titers against the remaining pathogens can only be explained by negative infection statuses with potential cross-reactivity from the present pathogen. Given that classic information criterion metrics such as the Akaike information criterion or deviance information criterion do not perform well in cases of mixture models of varying components, we instead used likelihood increment percentage (LIP) metrics to assess the relative improvements in model fit with increasing model components (Supplementary Materials and Methods) (31). Allowing each pathogen to be present in turn, we retained the present pathogen that resulted in the highest model log-likelihood before adding a second present pathogen. We repeated this process of adding present pathogens until the log-likelihood increment percentage per component (LIPpc) fell below 1%. We found the highest support for the presence of DENV1 in the study population, followed by CHIKV (LIPpc = 9.2%) and JEV (LIPpc = 5.0%) (Fig. 2A). Inclusion of a fourth present pathogen resulted in LIPpc values below 1%, and we therefore assumed that all of the remaining pathogens were absent from the study population. The selected present pathogens from this variable selection process were consistent across the three model versions considered, including a base model where a single value of prevalence is estimated by pathogen, a location-specific model where prevalence is estimated by subdistrict, and a location- and age-specific model where prevalence is estimated by subdistrict and age group (tables S1 to S3). We found that the location- and age-specific model version performed best by log-likelihood, and we therefore focus on the results of this model for the remainder of the manuscript. Model fit from this best-performing model is shown in fig. S2, and parameter estimates are shown in tables S4 to S6. In simulation studies, we also found that model versions that allow pathogen-specific prevalence to vary in the population (e.g., by location or age) are better able to accurately estimate true parameter values compared with those estimating a single population prevalence per pathogen (Supplementary Materials and Methods).

### Antibody profiles revealed endemic transmission of JEV and epidemics of DENV and CHIKV

Our final model estimated an overall prevalence of past infections of 17.0% [95% credible interval (95%CrI): 15.3 to 18.8%] for DENV1, 12.9% (95%CrI: 11.3 to 14.6%) for CHIKV, and 25.1% (95%CrI: 22.9 to 27.5%) for JEV in the study population (Fig. 2B). Reconstructing the most probable infection status of each individual, we estimated that 3.5% had been infected by both DENV1 and CHIKV, 2.5% by both DENV1 and JEV, 2.3% by both CHIKV and JEV, and 0.8% by all three. We found that pathogen-specific prevalence estimates did not substantially change as additional present pathogens were added (Fig. 2B). To understand how these estimates would compare with those derived from simpler analytical approaches that are traditionally used, we applied classic single-dimension (1D) mixture models and threshold approaches to antibody data independently for each pathogen. We found prevalence estimates for DENV1 and CHIKV to be consistent across models, whereas classic mixture model and threshold estimates of prevalence for DENV2, DENV4, TBEV, YFV, and ZIKV were close to zero, roughly aligned with inferences from our model framework. However, classic mixture models estimated a prevalence of 10.6% (95%CrI: 8.9 to 12.4%) for DENV3 and 7.5% (95%CrI: 5.7 to 9.5%) for WNV, inconsistent with our model framework, which found these antibody responses to be explained by cross-reactivity from DENV1 and JEV (Fig. 2B). In a simulation study, we further showed how bias in prevalence estimates obtained by classic 1D mixture models increased as both cross-reactivity from a related pathogen and prevalence of the related pathogen increased (Supplementary Materials and Methods).

Our model framework estimated substantial spatial variability in infection burden across the Chapai Nawabganj district. The prevalence of DENV1 ranged from 5.9% (4.0 to 8.3%) in Gomastapur in the north of the district to 30.3% (95%CrI: 25.8 to 34.6%) in Shibganj in the south (Fig. 2C). CHIKV prevalence was estimated to range from 4.4% (95%CrI: 2.1 to 7.8%) in Nachole to 28.4% (95%CrI: 24.3 to 32.7%) in Shibganj, consistent with where an outbreak had previously been detected (29). The prevalence of JEV infection ranged from 18.6% (95%CrI: 14.9 to 23.0%) in Shibganj to 33.7% (95%CrI: 27.4 to 40.4%) in Bholahat (Fig. 2C). Age-specific prevalence of DENV1 and CHIKV appeared constant among those ≥5 years of age (Fig. 2D). The lower prevalence estimated among 0- to 4-year-olds for DENV1 and CHIKV suggests the occurrence of an epidemic for each of these viruses sometime in the 5 years before the serological study (2009 to 2014). In contrast, we observed a pattern of increasing JEV prevalence by age in this district, indicative of endemic transmission (Fig. 2D). To characterize the transmission intensity of JEV, we fit simple catalytic models to age-specific prevalence estimates, assuming endemic transmission of JEV since its introduction to Bangladesh. Patterns in the age-specific prevalence estimates suggested the most likely JEV introduction date to be between 1965 and 1970 (Fig. 2D and fig. S3), roughly aligned with the first detected human cases in Bangladesh in 1977 (32, 33). We estimated that 1.2% (95%CrI: 1.1 to 1.3%) of the susceptible population in this region has been infected each year since JEV’s introduction, corresponding to ~19,179 (95%CrI: 17,347 to 21,128) annual JEV infections in the Chapai district (Fig. 2D). Estimates of transmission intensity of JEV varied from 0.8% (95%CrI: 0.6 to 1.1%) in Nawabganj Sadar to 1.7% (1.4 to 2.0%) in Gomastapur (fig. S4). We found our prevalence estimates to be consistent with or without inclusion of the DENV ELISA assay in model fitting (fig. S5). Estimates of JEV prevalence, however, were slightly higher when the DENV ELISA assay was included in model fitting, consistent with the lower sensitivity reported for the multiplex JEV EDIII antigen used in this study (30) and the high sensitivity of whole–E protein DENV ELISA assays.

### Cross-reactivity estimates align with genetic distances between antigens

**Fig. 3:**
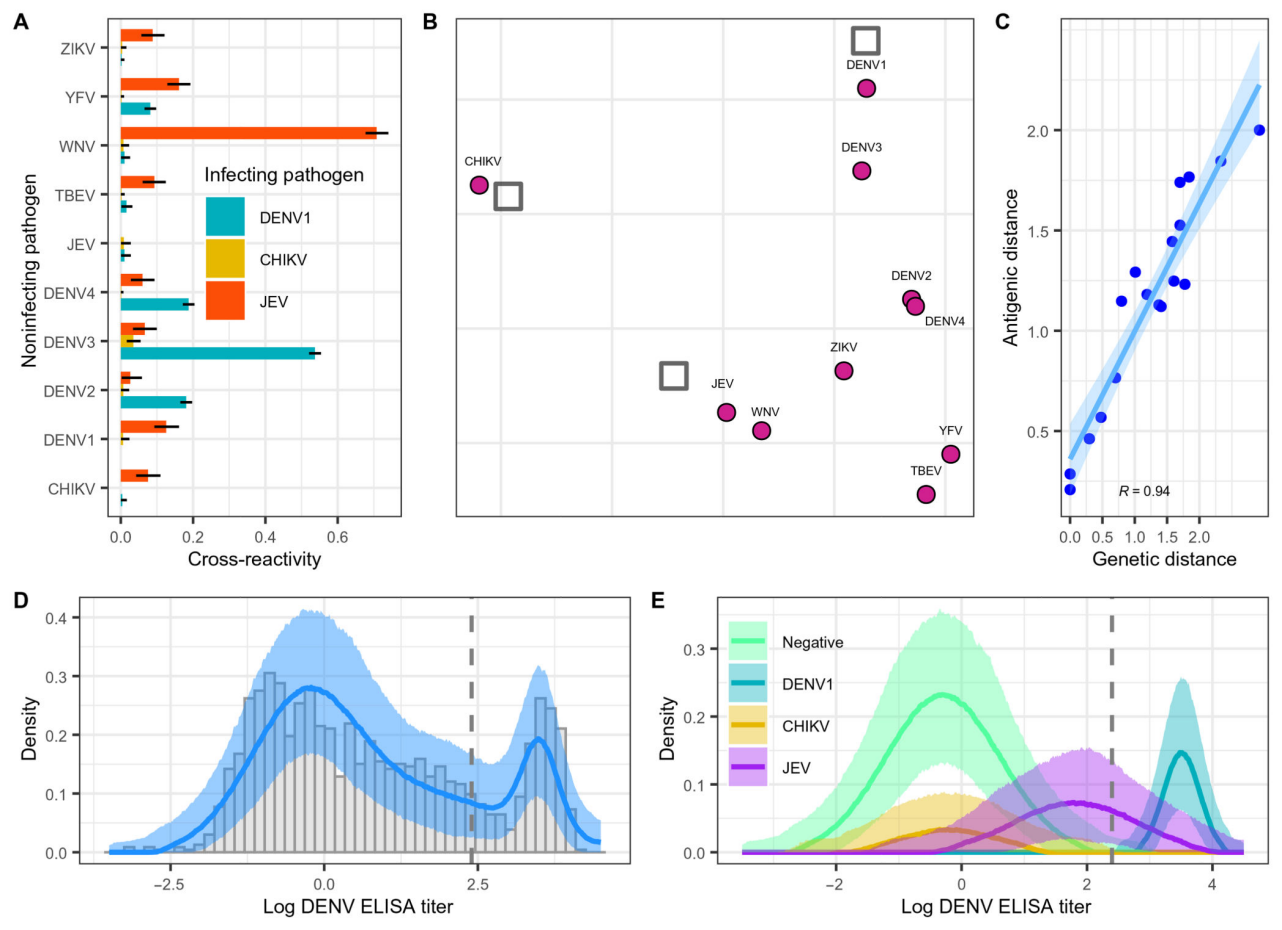
Antigenic relationships. (**A**) Model estimates of between-pathogen antibody cross-reactivity. Colored bars show the median model estimates of relative titer increases from each infecting pathogen against each noninfecting pathogen. Black lines show the 95%Crl estimates. (**B**) Antigenic cartography map of inferred antigenic distances between each serum sample (gray squares) representing binding IgG antibodies and antigen (purple circles), obtained by multidimensional scaling of the cross-reactivity estimates to a two-dimensional space. (**C**) Comparison of the inferred antigenic distances and genetic distances inferred using phylogenetic methods (blue points), considering only flavivirus pathogen pairs (i.e., excluding CHIKV), based on EDIII. The blue line and shaded ribbon show the mean and 95% confidence interval model fit of a linear regression with *r* = 0.94, respectively. (**D**) Observed (gray bars) and reconstructed log DENV ELISA population titer distributions, where the purple line and shaded ribbon show the model median and 95%Crl estimates, respectively. (**E**) Reconstructed log DENV ELISA titer distributions by infection status where colored lines and shaded ribbons indicate median and 95%Crl estimates, respectively. The gray dashed line in (D) and (E) shows the manufacturer-recommended threshold for defining DENV seropositivity.

From this best-performing model with three present pathogens, we estimated the highest antibody cross-reactivity from JEV to WNV with a relative titer increase of 0.71 (95%CrI: 0.68 to 0.74) against WNV among JEV-positive individuals (Fig. 3A). This implied that among those infected with JEV, an average of 71% of their response to the JEV antigen also bound to the WNV antigen. JEV infection was also estimated to induce a 0.16 (0.13 to 0.19) relative titer increase against YFV and a 0.13 (0.09 to 0.16) relative titer increase against DENV1. Among individuals with a past DENV1 infection, we estimated a 0.54 (95%CrI: 0.52 to 0.55) relative titer increase against DENV3 as well as 0.18 (0.16 to 0.20) and 0.19 (0.17 to 0.20) relative titer increases against DENV2 and DENV4, respectively (Fig. 3A). In contrast, we estimated minimal cross-reactivity from individuals with past CHIKV infection against the flavivirus antigens, with median estimates ranging from 0.00 to 0.03 (Fig. 3A). To summarize the antigenic relationships inferred by the model, we applied multidimensional scaling of the estimated antibody responses across infection statuses (Fig. 3B). We observed close antigenic clustering of the DENV serotypes, particularly DENV2 and DENV4, although the positions of these DENV antigens are informed only by the positive sera of a single DENV serotype (DENV1). We also inferred a close antigenic relationship between JEV and WNV, members of the same serocomplex (23), as well as between TBEV and YFV (Fig. 3B). To understand how our inferred antigenic distances compared with phylogenetic distances in the Flaviviridae family, we constructed a maximum-likelihood phylogeny of representative flavivirus EDIII proteins. We found high correlation between our inferred antigenic distances and genetic distances with Pearson *r* = 0.94 (Fig. 3C). The shortest genetic and antigenic distances were estimated between JEV and WNV and between DENV1 and DENV3, whereas the greatest antigenic and genetic distances were inferred between DENV1 and TBEV and between DENV1 and YFV (Fig. 3C).

To understand the relationship between each multiplex antigen and the more-sensitive DENV ELISA antigen, we estimated where the antibody titer distributions of each infection status fall on the DENV ELISA assay. The full reconstructed distribution of DENV ELISA titers is shown in Fig. 3D. Of the present pathogens included in our model, we estimated that DENV1-positive individuals had the highest response on the DENV ELISA assay with mean titers of 3.5 (95%CrI: 3.5 to 3.6), shown in Fig. 3E. We also estimated a substantial signal from individuals with past JEV infection to the DENV ELISA assay, with mean titers of 1.8 (95%CrI: 1.7 to 1.9). In contrast, individuals with past CHIKV infection had mean DENV ELISA titers of −0.2 (95%CrI: −0.4 to 0.0), similar to those of individuals negative for all pathogens (mean DENV ELISA titer −0.3; 95%CrI: −0.4 to −0.2) (Fig. 3E). Using the manufacturer-recommended cut-off for defining DENV seropositivity resulted in a prevalence of 22.0% (95%CI: 19.9 to 24.3%), slightly higher than our estimates of DENV1 prevalence of 17.0% (95%CrI: 15.3 to 18.8%), inferred by the full model. Results from our model indicated that the difference in estimates is attributed to individuals previously infected with JEV who have high responses on the DENV ELISA assay (Fig. 3E), giving the appearance of increasing DENV ELISA titers by age (fig. S6). The DENV ELISA assay uses the whole E protein and is therefore more sensitive to cross-reactivity compared with the EDIII used in the multiplex assay. Taking the DENV ELISA results on their own could easily be misinterpreted as endemic transmission of DENV.

### Cross-reactive signals allow detection of unobserved pathogens

**Fig. 4:**
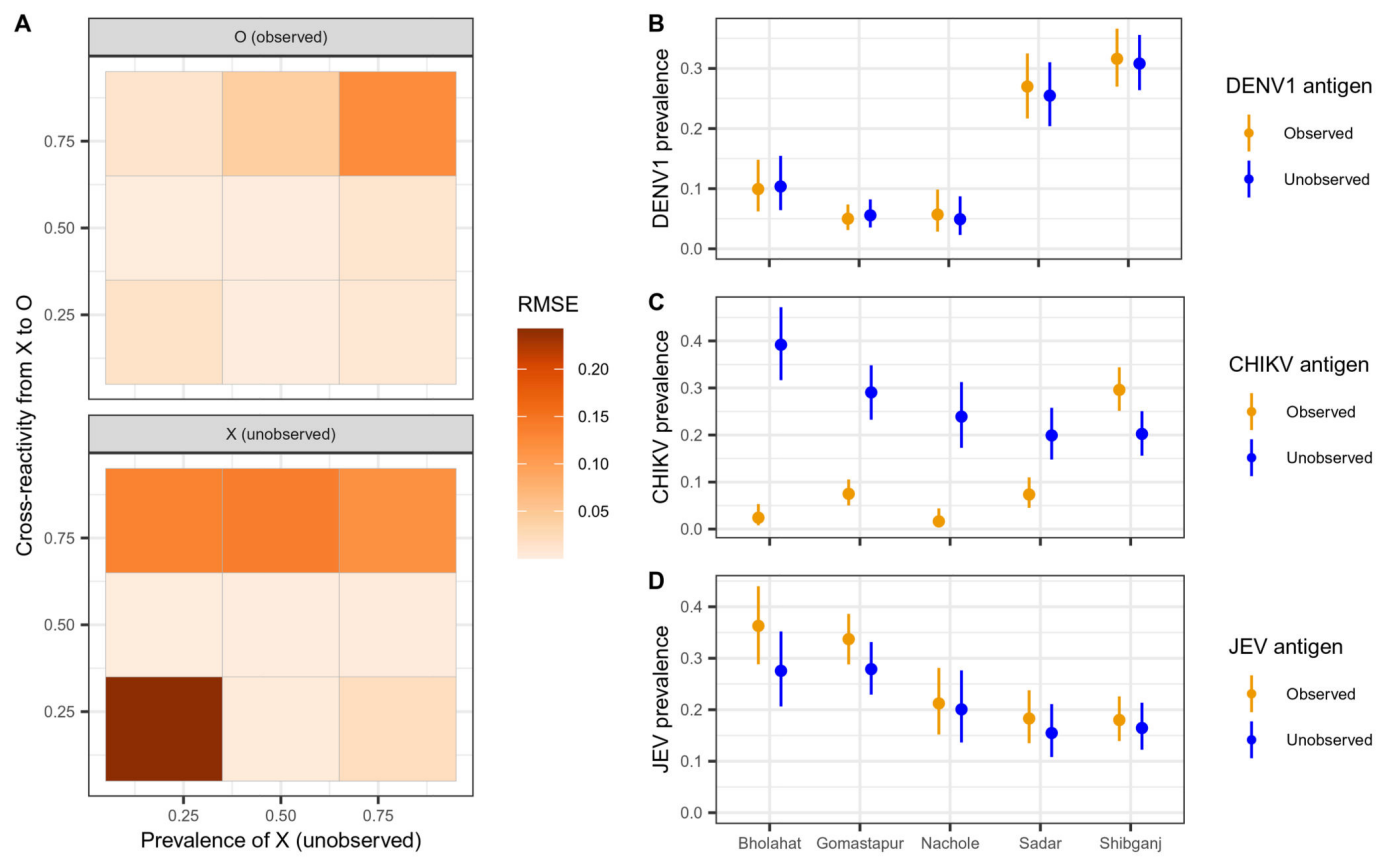
Detecting unobserved pathogens. (**A**) Heatmap of the RMSE of prevalence estimates obtained from simulated data for an observed pathogen, O, and an unobserved pathogen, X, with varying cross-reactivity from X to O and varying prevalence of the unobserved pathogen X. (**B** to **D**) Comparisons of model estimates of pathogen prevalence when antibody titers against the homologous antigen are observed and included in the model (orange) and when they are excluded/unobserved but reconstructed as an unobserved pathogen X. Results are shown for DENV (B), CHIKV (C), and JEV (D). Points and lines indicate model median and 95%Crl estimates, respectively.

A key uncertainty that arises in multipathogen serological studies is the potential presence of cross-reactive antibodies induced from unmeasured or unknown pathogens. This raises the potential issue of misattributing antibody responses to related pathogens that have been tested for. We therefore used our analytical method to explore the scenarios in which the presence and prevalence of an unobserved pathogen can be inferred through cross-reactive antibody responses. We conducted simulations of a three-pathogen system, where two pathogens are observed or tested for and one is not. We found that estimates of the prevalence of observed pathogens (O) and the unobserved pathogen (X) can both be accurately inferred at intermediate values of cross-reactivity from X to O, with a low root mean square error (RMSE) (Fig. 4A). At lower values of cross-reactivity from X to O, the prevalence of observed pathogens O can be estimated with good accuracy because of limited interference from X. However, the prevalence of X cannot be well identified when its true prevalence and its cross-reactivity to observed pathogens are both low. Moreover, at higher values of cross-reactivity from X to O, the prevalence of X cannot be accurately reconstructed, with the prevalence of O also becoming unidentifiable as the prevalence of X increases (Fig. 4A).

To assess the generalizability of these findings to real data, we refit our final model to the arbovirus antigens, excluding 1 of the 10 antigens and attempting to reconstruct the parameters for this unobserved pathogen. We showed that our model was able to accurately reconstruct the prevalence of both DENV1 and JEV as unobserved pathogens when antibody measurements from these antigens were excluded from model fitting (Fig. 4, B to D). In addition, the model accurately reconstructed estimates of cross-reactivity from the unobserved pathogens to each observed antigen, including close antigenic relationships between JEV and WNV and between DENV1 and DENV3 (fig. S7). In practice, if the DENV1 or JEV antigens are missing, we may likely conclude that DENV3 (instead of DENV1) or WNV (instead of JEV) is present instead. In contrast with the case of DENV1 and JEV, we showed that when the CHIKV antigen is excluded from fitting, the model cannot accurately reconstruct the parameters of CHIKV. This is consistent with the simulation results that demonstrated that the lack of cross-reactivity between CHIKV and flavivirus antigens inhibits its identification, whereas intermediate cross-reactivity between JEV and DENV1 with the other flavivirus antigens enables their accurate reconstruction.

## Discussion

The transmission of multiple antigenically related pathogens in a population has long posed a challenge to the interpretation of serological studies of flaviviruses and other antigenically variable pathogens. In contexts where multiple related, cross-reacting pathogens have been transmitted in a population, it can be difficult to understand whether antibodies detected against a specific pathogen were induced through exposure to that pathogen itself or to a related pathogen. In this study, we applied an analytical framework to multipathogen serological data to disentangle the multidimensional antigen-antibody relationships. We used this framework to infer estimates of arboviral prevalence in northwest Bangladesh by location and age, accounting for varying degrees of between-pathogen antibody cross-reactivity.

A key question arising from multipathogen serological studies is determining which pathogens have truly transmitted in the study population. To date in Bangladesh, there has been no evidence of human transmission for YFV or TBEV, whereas human cases of DENV, CHIKV, JEV, and, more recently, ZIKV and WNV have all been reported in the country (28, 29, 32, 34–36). Among the pathogens included in the study, we found evidence of past DENV, CHIKV, and JEV transmission in the district of Chapai Nawabganj, whereas no strong evidence for the presence of the remaining pathogens was found. Our prevalence estimates for this district (17, 13, and 25% for DENV, CHIKV, and JEV, respectively) are lower than national estimates for DENV (24%) but higher than national estimates for CHIKV (2.4%) and JEV (3.4%) (7, 37, 38). We showed that simpler methods that do not account for antibody cross-reactivity would have inferred the presence of WNV, whereas, in our model, WNV antibodies were found to be explained by high cross-reactivity from JEV infections. This is consistent with local epidemiological data, with confirmed JEV hospitalized cases regularly reported in this district (28, 33). In contrast, the first diagnosed human case of WNV in Bangladesh was reported in 2019 in Dhaka, 5 years after the completion of the serological study (36). We inferred the highest prevalence of DENV and CHIKV in the more urbanized district of Shibganj, where a past epidemic of CHIKV had been reported, consistent with the *Aedes* vector’s ability to thrive in urban environments (29). For ZIKV, the first detected human case in Bangladesh was in August 2014, with serological evidence of ZIKV infections in Dhaka since 2013 (34, 39). However, we found no evidence of ZIKV transmission in the district of Chapai Nawabganj as of 2014.

Our analysis explored how a combination of sensitive and specific antigens can be leveraged as a means of detecting the presence and prevalence of unknown or unmeasured pathogens. We demonstrated how signals from related cross-reacting pathogens could make the detection of unobserved pathogens possible under conditions of intermediate cross-reactivity profiles. An important limitation to this is that the relationship of the unobserved pathogen to the measured pathogens, and therefore the reliability of inference, will not be known in many real-world applications. Future work to define robust criteria or strength of evidence for the detection of unobserved pathogens through serological surveillance will be important for public health applications. Routine monitoring of population serological profiles over time could allow real-time detection of previously unidentified pathogens through shifts in baseline cross-reactive profiles. The major emerging pathogens of recent years have all belonged to families of known human pathogens, with coronaviruses, flaviviruses, and influenza viruses being the predominant examples. Our findings highlight how emerging pathogens of known pathogen families could be detected through routine serological testing, supporting the potential benefits of a global immunological observatory and routine serological surveillance (40, 41).

There are a number of limitations that should be considered when interpreting the results of our analyses. Antibody kinetics are not accounted for in our model framework, which relies on cross-sectional serological data representing a snapshot of population antibody titers at a single point in time. Because the timing and sequence of infections cannot be accounted for, our model estimates will therefore reflect population averages across a range of time-since-infection scenarios represented in the study population. If antibodies wane substantially after infection, then the prevalence of infection may be underestimated. Our approach also assumes lifelong homotypic immunity against reinfection and no postinfection boosting. However, in the case of the arboviruses measured in this study, infection is thought to induce long-term homotypic IgG responses (42, 43). In addition, if an infection induces higher antibody responses against a related pathogen than the infecting pathogen, then the infection statuses may be misclassified. We assume that cross-reactive antibodies from infecting pathogens to any non-infecting pathogen combine additively, which may not hold for individuals infected with an increasing number of related pathogens. Our model represents an unsupervised approach to classifying population infection statuses, which has the advantage of not relying on validated sample sets that can be time- and resource-intensive to obtain. Although our extensive simulation study demonstrated the theoretical validity of our analytical framework, future analysis from serological cohort studies where infections are confirmed by polymerase chain reaction or other methods will allow for an improved understanding of model performance.

Despite these limitations, the antigenic distances between assay antigens and monotypic sera inferred by our model were found to be highly consistent with genetic distances between antigens independently inferred from phylogenetic methods. In addition, our inferences of arbovirus infection burden are consistent with known epidemiological indicators of each present pathogen. Previous studies have analyzed serological data considering two related pathogens using additional assumptions of pathogen transmission dynamics (25, 26). The analytical framework presented here is agnostic to any specific pathogen transmission dynamics, allowing it to scale to many pathogens with potentially different transmission dynamics. This framework has been shown to provide robust estimates of the prevalence of past infections in systems of several related pathogens while simultaneously accounting for varying amounts of between-pathogen cross-reactivity. In this study, we demonstrated the utility of this analytical approach to making robust epidemiological inferences of public health relevance. This framework could disentangle high-dimensional antibody-antigen relationships of multiple related pathogens, providing insights into the burden and transmission dynamics, as well as the immune landscape, of arboviral diseases.

## Materials and Methods

### Study design

We analyzed data from a cross-sectional seroprevalence study conducted in 2014 in the Chapai Nawabganj zila (district) of Bangladesh, located in the Rajshahi division. A total of 1453 individuals were included in the study from 39 communities across the five upazilas (subdistricts) of Chapai Nawabganj. Participant recruitment in each rural community was initiated by identifying the household where the most recent wedding took place and beginning recruitment in the house of their closest neighbor. In each urban community, recruitment was initiated by finding the nearest community center and identifying the closest neighboring household for participant recruitment. All residents of selected houses, regardless of age, were eligible for enrollment in the study. After the first enrolled house-hold, the next five closest neighbors were skipped, and the sixth closest household was approached for enrollment. This process was repeated until individuals from at least 15 different households were enrolled from that community. Individuals unable to give consent because of disability or who had an acute medical condition where blood collection is contraindicated were excluded from study participation. A single 5-ml venous blood sample was collected from each enrolled participant. The age distribution of the study participants compared with the population age distribution for each of the five upazilas is shown in fig. S8. Data on the population age distributions were obtained from the 2011 Bangladesh census (44). The serological study was approved by the ethical review boards at the US Centers for Disease Control and Prevention; International Centre for Diarrhoeal Disease Research, Bangladesh (PR-13001); Johns Hopkins Bloomberg School of Public Health; and Institut Pasteur. Informed consent, and assent when appropriate, was obtained from all study participants.

### Multiplex immunoassay

Serum samples were tested for the presence of IgG antibodies against 10 antigens using an in-house microsphere-based multiplex immunoassay. Recombinant EDIII antigens were used for all flavivirus pathogens (DENV1, DENV2, DENV3, DENV4, JEV, WNV, TBEV, YFV, and ZIKV) to improve assay sensitivity and specificity, as previously described (30), and a recombinant E2 glycoprotein was used for CHIKV. The assay was previously validated using 172 negative and 300 positive control sera from horses infected with WNV, JEV, and TBEV (30). Magnetic beads were coupled to each specific antigen, and the assay was conducted as previously described, including determining individual-level background controls using a recombinant human protein SNAP-tag (O^6^-methylguanine DNA methyl-transferase) (30). Fluorescence intensity and bead color coding were measured using a Luminex 200 system (Bio-Rad Laboratories). For each sample, an RFI value was calculated by dividing the median fluorescence intensity by the fluorescence intensity of the individual-level background control. All analyses of multiplex immunoassay data were conducted using log RFI values. In addition to the 10 antigens measured with the multiplex immunoassay, a Panbio DENV ELISA assay using the whole DENV E protein was also used to measure binding DENV IgG antibody concentrations.

### Multivariate mixture model

We developed and applied a semimechanistic multivariate Gaussian mixture model to jointly infer the prevalence of infection of multiple pathogens and between-pathogen antibody cross-reactivity. We assumed the long-term persistence of IgG antibodies after infection and therefore defined positivity on the basis of the presence or absence of serological evidence of a past infection. We considered a system of *n = P* pathogens, where the true infection status of each individual is either negative, (0), or positive, (1), for each pathogen. For a system of *P* pathogens with two possible outcomes, there are 2^*P*^ possible infection status combinations. For each possible infection status combination, *c*, we define a *P*-dimensional Gaussian component, with vector of means μ_*c*_ and covariance matrix Σ_*c*_. The proportion of the study population with infection status combination *c*, θ_*c*_, was defined as the conditional probability of having infection status combination *c*, given the pathogen-specific population prevalence, π_*p*_. For example, for infection status combination *c* = {0,1,0} where individuals have been infected by pathogen B only, the proportion of the population with this infection status is calculated as θ_*c*_ = (1 −π_*A*_) (π_*B*_) (1 − π_*C*_).

We first considered a system of two related pathogens, A and B. For each of the four possible infection status combinations, we defined a bivariate Gaussian component to characterize the antibody titers of this status. For individuals negative for both A and B, {*A*_0_, *B*_0_}, the Gaussian component is defined with means $\mu_{A_{0}}$ and $\mu_{B_{0}}$ and standard deviations $\sigma_{A_{0}}$ and $\sigma_{B_{0}}$. We allowed for correlation in these negative antibody titers, with correlation coefficient ρ_0_. The covariance matrix, Σ, for this Gaussian component was then defined as shown in Eq. 1, where the covariance of A and B is $\text{Cov}\left(A_{0},B_{0}\right)=\rho_{0}\sigma_{A_{0}}\sigma_{B_{0}}$

(1) $$\Sigma \left\{A_{0},B_{0}\right\}=\left(\begin{array}{cc} \sigma_{A_{0}}^{2} & \rho_{0}\sigma_{A_{0}}\sigma_{B_{0}}\\ \rho_{0}\sigma_{A_{0}}\sigma_{B_{0}} & \sigma_{B_{0}}^{2} \end{array}\right)$$

For individuals infected with pathogen B only, {*A*_0_, *B*_1_}, we defined the mean rise in antibody titers against pathogen B as $\mu_{B_{R}}$ such that the mean antibody titers against pathogen B are $\mu_{B_{1}}=\mu_{B_{0}}+\mu_{B_{R}}$, with standard deviation $\sigma_{B_{1}}$. We assumed that this bivariate Gaussian component was a linear combination of negative antibody titers against pathogen A and positive antibody titers against pathogen B. In this way, the mean antibody titers against pathogen A were calculated as $\mu_{A_{0}}+\phi_{BA}\mu_{B_{1}}$. Here, ϕ_*BA*_ is the relative increase in antibody titers raised against pathogen A after infection with pathogen B relative to the antibody titers binding to pathogen B. The covariance in antibody titers for this Gaussian component was calculated as shown in Eqs. 2 and 3 using the bilinearity property of covariance. Here, we assumed that the covariance in these antibody titers in the absence of cross-reactivity was zero, Cov (*A*_0_, *B*_1_ = 0). Under this assumption, the standard deviation for antibody titers against pathogen A can be calculated as $\sqrt{\sigma_{A_{0}}^{2}+{\left(\phi_{BA}\sigma_{B_{1}}\right)}^{2}}$. The covariance matrix for this bivariate Gaussian component can subsequently be defined as shown in Eq. 4

(2) $$\text{Cov}\left(A_{0}+\phi_{BA}B_{1},B_{1}\right)=\text{Cov}\left(A_{0},B_{1}\right)+\phi_{BA}\text{Cov}\left(B_{1},B_{1}\right)$$

(3) $$\text{Cov}\left(A_{0}+\phi_{BA}B_{1},B_{1}\right)=\phi_{BA}\sigma_{B_{1}}^{2}$$

(4) $$\Sigma \left\{A_{0},B_{1}\right\}=\left(\begin{array}{cc} \sigma_{A_{0}}^{2}+{\left(\phi_{BA}\sigma_{B_{1}}\right)}^{2} & \phi_{BA}\sigma_{B_{1}}^{2}\\ \phi_{BA}\sigma_{B_{1}}^{2} & \sigma_{B_{1}}^{2} \end{array}\right)$$

For individuals infected with A only, {*A*_1_, *B*_0_}, the bivariate Gaussian component was defined with the same approach as before. The Gaussian means were defined as $\mu_{A_{0}}+\mu_{A_{R}}$ and $\mu_{B_{0}}+\phi_{AB}\mu_{A_{1}}$, respectively, for antibody titers against pathogens A and B, and the standard deviations were defined as $\sigma_{A_{1}}$ and $\sqrt{\sigma_{B_{0}}^{2}+{\left(\phi_{AB}\sigma_{A_{1}}\right)}^{2}}$. With the same approach as above (Eqs. 2 and 3), the covariance matrix of this Gaussian component was defined as shown in Eq. 5. We assumed that cross-reactive antibodies cannot decrease antibody titers, i.e., ϕ ≥ 0. In addition, we allowed for asymmetric cross-reactive antibody responses such that ϕ_*BA*_ is independent of ϕ_*AB*_

(5) $$\Sigma \left\{A_{1},B_{0}\right\}=\left(\begin{array}{cc} \sigma_{A_{1}}^{2} & \phi_{AB}\sigma_{A_{1}}^{2}\\ \phi_{AB}\sigma_{A_{1}}^{2} & \sigma_{B_{0}}^{2}+{\left(\phi_{AB}\sigma_{A_{1}}\right)}^{2} \end{array}\right)$$

Last, for the infection status combination {*A*_1_, *B*_0_}, where individuals have been infected with both pathogens A and B, we defined the means of the Gaussian component as $\mu_{A_{1}}=\mu_{A_{0}}+\mu_{A_{R}}$ and $\mu_{B_{1}}=\mu_{B_{0}}+\mu_{B_{R}}$. Without knowing the sequence or timing of infections, we assumed that the means and standard deviations of positive antibody titers were constant across infection statuses. Therefore, the standard deviations of this Gaussian component were simply defined as $\sigma_{A_{1}}$ and $\sigma_{B_{1}}$ with correlation coefficient ρ_1_. The covariance matrix for this Gaussian component was then defined as shown in Eq. 6

(6) $$\Sigma \left\{A_{1},B_{1}\right\}=\left(\begin{array}{cc} \sigma_{A_{1}}^{2} & \rho_{1}\sigma_{A_{1}}\sigma_{B_{1}}\\ \rho_{1}\sigma_{A_{1}}\sigma_{B_{1}} & \sigma_{B_{1}}^{2} \end{array}\right)$$

More generally, for a system with *n* = *P* pathogens, the number of possible infection status combinations, and therefore the number of *P*-dimensional Gaussian components used to describe the data, is 2^*P*^. To reduce the number of new parameters required when scaling the system to higher dimensions, we assumed that correlation in antibody titers when individuals are negative to all pathogens, ρ_0_, is constant across pathogen pairs. For instance, the covariance matrix for infection status {*A*_0_, *B*_0_, *C*_0_} in a three-pathogen system is defined as shown in Eq. 7.

(7) $$\Sigma \left\{A_{0},B_{0},C_{0}\right\}=\left(\begin{array}{ccc} \sigma_{A_{0}}^{2} & \rho_{0}\sigma_{A_{0}}\sigma_{B_{0}} & \rho_{0}\sigma_{A_{0}}\sigma_{C_{0}}\\ \rho_{0}\sigma_{A_{0}}\sigma_{B_{0}} & \sigma_{B_{0}}^{2} & \rho_{0}\sigma_{B_{0}}\sigma_{C_{0}}\\ \rho_{0}\sigma_{A_{0}}\sigma_{C_{0}} & \rho_{0}\sigma_{B_{0}}\sigma_{C_{0}} & \sigma_{C_{0}}^{2} \end{array}\right)$$

For infection statuses where individuals have been infected with one pathogen, the covariance in antibody titers against the infecting pathogen and noninfecting pathogens was calculated with the same approach as before (Eqs. 2 and 3). The covariance in antibody titers for pairs of noninfecting pathogens, now in the presence of cross-reactive antibodies from a related pathogen, was calculated as shown in Eqs. 8 to 10, taking the example of infection status {*A*_0_, *B*_0_, *C*_1_}, where individuals are positive for pathogen C only. As before, we assumed antibody titers against infecting and noninfecting pathogens to be independent in the absence of cross-reactivity such that Cov (*A*_0_, *C*_1_) = 0 and Cov (*B*_0_, *C*_1_) = 0. The covariance matrix for this infection status is then defined as shown in Eq. 11

(8) $$\text{Cov}\left(A_{0},B_{0}|C_{1}\right)=\text{Cov}\left(A_{0}+\phi_{CA}C_{1},B_{0}+\phi_{CB}C_{1}\right)$$

(9) $$\text{Cov}\left(A_{0},B_{0}|C_{1}\right)=\text{Cov}\left(A_{0},B_{0}\right)+\phi_{CB}\text{Cov}\left(A_{0},C_{1}\right)+\phi_{CA}\text{Cov}\left(C_{1},B_{0}\right)+\phi_{CA}\phi_{CB}\text{Cov}\left(C_{1},C_{1}\right)$$

(10) $$\text{Cov}\left(A_{0},B_{0}|C_{1}\right)=\rho_{0}\sigma_{A_{0}}\sigma_{B_{0}}+\phi_{CA}\phi_{CB}\sigma_{C_{1}}^{2}$$

(11) $$\Sigma \left\{A_{0},B_{0},C_{1}\right\}=\left(\begin{array}{ccc} \sigma_{A_{0}}^{2}+{\left(\phi_{CA}\sigma_{C_{1}}\right)}^{2} & \rho_{0}\sigma_{A_{0}}\sigma_{B_{0}}+\phi_{CA}\phi_{CB}\sigma_{C_{1}}^{2} & \phi_{CA}\sigma_{C_{1}}^{2}\\ \rho_{0}\sigma_{A_{0}}\sigma_{B_{0}}+\phi_{CA}\phi_{CB}\sigma_{C_{1}}^{2} & \sigma_{B_{0}}^{2}+{\left(\phi_{CB}\sigma_{C_{1}}\right)}^{2} & \phi_{CB}\sigma_{C_{1}}^{2}\\ \phi_{CA}\sigma_{C_{1}}^{2} & \phi_{CB}\sigma_{C_{1}}^{2} & \sigma_{C_{1}}^{2} \end{array}\right)$$

In the case of infection status combinations where individuals have been infected with more than one pathogen, we assumed that the means and standard deviations of cross-reactive antibody titers against noninfecting pathogens increased additively. For instance, for an infection status {*A*_0_, *B*_1_, *C*_1_}, the mean of the Gaussian component characterizing titers against pathogen A was calculated as $\mu_{A_{0}}+\phi_{BA}\mu_{B_{1}}+\phi_{CA}\mu_{C_{1}}$. Similarly, the standard deviation for antibody titers against pathogen A was calculated as $\sqrt{\sigma_{A_{0}}^{2}+{\left(\phi_{BA}\sigma_{B_{1}}\right)}^{2}+{\left(\phi_{CA}\sigma_{C_{1}}\right)}^{2}}$. The covariance between negative and positive titers was calculated as shown in Eq. 12, additionally accounting for the correlation in antibody titers for the positive pathogens, ρ_1_. We assumed that this correlation in antibody titers between pairs of positive pathogens was constant across all pathogen pairs and infection status combinations. The covariance matrix for infection status {*A*_0_, *B*_1_, *C*_1_} is shown in Eq. 13

(12) $$\text{Cov}\left(A_{0},B_{1}|C_{1}\right)=\phi_{BA}\sigma_{B_{1}}^{2}+\phi_{CA}\rho_{1}\sigma_{B_{1}}\sigma_{C_{1}}$$

(13) $$\Sigma \left\{A_{0},B_{1},C_{1}\right\}=\left(\begin{array}{ccc} \sigma_{A_{0}}^{2}+{\left(\phi_{BA}\sigma_{B_{1}}\right)}^{2}+{\left(\phi_{CA}\sigma_{C_{1}}\right)}^{2} & \phi_{BA}\sigma_{B_{1}}^{2}+\phi_{CA}\rho_{1}\sigma_{B_{1}}\sigma_{C_{1}} & \phi_{CA}\sigma_{C_{1}}^{2}\\ \phi_{BA}\sigma_{B_{1}}^{2}+\phi_{CA}\rho_{1}\sigma_{B_{1}}\sigma_{C_{1}} & \sigma_{B_{1}}^{2} & \rho_{1}\sigma_{B_{1}}\sigma_{C_{1}}\\ \phi_{CA}\sigma_{C_{1}}^{2} & \rho_{1}\sigma_{B_{1}}\sigma_{C_{1}} & \sigma_{C_{1}}^{2} \end{array}\right)$$

More generally, in the case of covariance in antibody titers against pairs of noninfecting pathogens, in the presence of >1 infecting pathogen, the covariance was calculated as shown in Eq. 14, with *K* total pathogens. In the case of covariance in antibody titers for infecting and noninfecting pathogens, in the presence of >1 infecting pathogen, the covariance was calculated as shown in Eq. 15

(14) $$\text{Cov}\left(A_{0},B_{0}|C_{1},D_{1},\ldots K_{1}\right)=\rho_{0}\sigma_{A_{0}}\sigma_{B_{0}}+\sum_{n=C}^{K}\phi_{nA}\phi_{nB}\sigma_{n_{1}}^{2}+\sum_{n=C}^{K-1}\sum_{m=n+1}^{K}\left(\phi_{nA}\phi_{mB}+\phi_{mA}\phi_{nB}\right)\rho_{0}\sigma_{0}^{2}$$

(15) $$\text{Cov}\left(A_{0},B_{1}|C_{1},D_{1},\ldots K_{1}\right)=\phi_{BA}\sigma_{B_{1}}^{2}+\sum_{n=C}^{K}\phi_{nA}\rho_{1}\sigma_{B_{1}}\sigma_{n_{1}}$$

### Present versus absent pathogens

To understand which pathogens are likely to have transmitted in the population, we conducted a stepwise variable selection process, comparing the performance of models that assumed a pathogen to be present versus absent. In the case of pathogens that are assumed to be absent from the study population, the model can be simplified by reducing the number of possible infection status combinations and therefore the number of Gaussian components needed to describe the data. For instance, considering a system of three pathogens, A, B, and C, there are eight possible infection status combinations if all three pathogens have transmitted in the population. In contrast, if pathogen C can be assumed to be absent from the study population (i.e., has never transmitted in the population), then the number of infection status combinations is halved. In this way, the antibody titers against the absent pathogen were still included in model fitting and were characterized only by the negative cross-reactivity Gaussian components/infection statuses. The number of parameters was also reduced, with π and μ_1_ parameters no longer estimated for each absent pathogen. Cross-reactivity parameters ϕ from the absent pathogens to the present pathogens were also not estimated, but cross-reactivity from the present pathogens to the absent ones was estimated.

### Unobserved pathogens

We explored the ability of the model framework to accurately reconstruct the prevalence of past infection of some unmeasured/unobserved pathogen, “pathogen X,” that had not been tested for by the serological assay. Additional Gaussian components were fit to characterize the antibody titer distributions of pathogen X, with 2^*P*+1^ possible infection status combinations. Covariance matrices for each infection status combination were of size *P*∗*P*, where *P* is the number of observed pathogens. The prevalence of the unmeasured pathogen was estimated, as was the cross-reactivity from the unobserved pathogens to the measured pathogens. Because the homologous binding to pathogen X is unknown, we assumed some fixed value of mean titers against pathogen X from which the relative titer increase against other pathogens could be inferred. We assumed that the standard deviation of these positive titers against pathogen X was equal to the standard deviation of positive responses against the measured pathogens.

### Model fitting

We applied the model framework to the arbovirus serological data from Chapai Nawabganj. We considered the data as being representative of the 10 pathogens that were tested using the multiplex immunoassay (CHIKV, DENV1, DENV2, DENV3, DENV4, JEV, TBEV, WNV, YFV, and ZIKV) and an additional antigen (DENV ELISA assay), giving a total of 11 antigens. We assumed that the standard deviations of negative antibody responses, σ_0_, and positive antibody responses, σ_1_, were each constant across the multiplex immunoassay antigens. We also assumed that correlation in antibody responses against pairs of infecting pathogens, ρ_0_, was 0. We fit the multivariate mixture model framework jointly to data from all 11 antigens and conducted a stepwise variable selection process to identify which of the 10 pathogens were likely to be present in the study population. We considered a “base” model version that estimates a single prevalence per pathogen, as well as location-specific and location- and age-specific versions. In the location-specific versions, pathogen-specific prevalence was allowed to vary by each of the five subdistricts of Chapai Nawabganj. For the location- and age-specific version, we further allowed pathogen prevalence to vary by age group. We assumed all other parameters to be constant across the study population, allowing only pathogen-specific prevalence to vary in the population. Model run times with increasing present pathogens are shown in fig. S9. All code used for model fitting is available at https://doi.org/10.5281/zenodo.17340210, with additional resources at https://github.com/meganodris/MultiSero.

### Variable selection process

We started by assuming that only 1 of the 10 pathogens was present in the study population such that antibody titers for the remaining 9 pathogens must be explained by negative infection statuses and cross-reactive antibodies from the single present pathogen. We fit this model, assuming each of the 10 pathogens to be present in turn, and identified the model with the highest log-likelihood from this step. The present pathogen from the best-performing model was then retained in the next step, where a second present pathogen was added. We repeated this iterative process, at each step identifying the additional present pathogen that improved the likelihood the most. We continued the process until the LIPpc fell below a threshold of 1%. Because of the limitations of traditional information criterion approaches for the comparison of finite mixture models of varying complexity, we focused on the LIP metric (31, 45). We used the LIPpc to assess the improvement in model fit across models with an increasing number of present pathogens and therefore increasing numbers of Gaussian components used to explain the data. Here, the increase in log-likelihood for model *j* (logLik_*j*_), relative to the log-likelihood of simpler model *j* – 1, (logLik_*j*–1_), was considered, accounting for the additional Gaussian components, *g*, fitted by model *j*, *g*_*j*_, as shown in Eq. 16

(16) $${\text{LIPpc}}_{j}=\frac{100\left(1-\frac{-2{\text{logLik}}_{j}}{-2{\text{logLik}}_{j-1}}\right)}{g_{j}-g_{j-1}}$$

### DENV ELISA assay

We included the DENV ELISA as an additional antigen/dimension in the full model framework. Because of the inclusion of DENV antigens in the multiplex immunoassay, we did not consider the DENV ELISA to be an independent pathogen that can be present. We instead allowed the DENV ELISA antibody titers to be explained by the same Gaussian components describing the multiplex immunoassay antibody responses. We assumed no covariance between antibody titers measured on the DENV ELISA and on the multiplex immunoassay. We estimated the mean, μ_*E*_, and standard deviation, σ_*E*_, of DENV ELISA titers for infection status combinations that were negative for all pathogens and those positive for a single pathogen. For infection statuses that are positive for >1 pathogen, we assumed that the mean DENV ELISA titers were equal to the maximum μ_*E*_ of the Gaussian components describing the respective monotypic infection statuses. The standard deviation of DENV ELISA titers for an infection status positive to >1 pathogen was calculated as the joint standard deviation of the respective monotypic infection statuses. For example, the standard deviation of DENV ELISA titers for an infection status positive for pathogens A and B, $\sigma_{E_{A1B1}}$, was calculated as $\sqrt{\sigma_{E_{A1}}^{2}+\sigma_{E_{B1}}^{2}}$.

### Reconstructing unobserved pathogens

To test the ability of our model to reconstruct the prevalence of past infection of an unobserved pathogen, we excluded antibody titer data from a single pathogen from model fitting. In particular, of the pathogens that were determined to be present in the study population from the variable selection process, we excluded the multiplex antigen of each in turn. We included the DENV ELISA assay in model fitting and inferred the prevalence of each observed and unobserved pathogen by subdistrict. We assumed a fixed value of 2 for mean positive titer responses against pathogen X for inference of the cross-reactivity responses from pathogen X to observed pathogens.

### Likelihood and priors

We assumed that the antibody titers for each infection status combination followed a multivariate Gaussian distribution, with the probability of observing antibody titers *x* for individual *i*, given infection status combination *c*, as shown in Eq. 17. Here, *x* was an *n* = *P* vector of antibody titer measurements to each pathogen, and 𝒩 represents a probability density function of a normal (Gaussian) distribution. The full model log-likelihood was calculated as the sum of the log probabilities across individuals, *i*, and across all possible infection status combinations, *c*, weighted by the proportion of the study population with infection status combination *c*, θ_*c*_, as shown in Eq. 18. The log-likelihood of the location and age-specific model was calculated similarly to before, as shown in Eq. 19, but now with location- and age-specific θ_*c*,*l*,*a*_ derived from the location- and age-specific prevalence estimates π_*p*,*l*,*a*_, as previously described

(17) $$N\left(x|\mu_{c},\Sigma_{c}\right)=\frac{1}{2\pi^{P/2}{\left|\Sigma_{c}\right|}^{1/2}}\text{exp}\left(-\frac{1}{2}{\left(x-\mu_{c}\right)}^{T}\Sigma_{c}^{-1}\left(x-\mu_{c}\right)\right)$$

(18) $$\text{logLik}=\overset{i}{\sum }\ln\overset{c}{\sum }\theta_{c}N\left(x_{i}|\mu_{c},\Sigma_{c}\right)$$

(19) $$\text{logLik}=\overset{i}{\sum }\ln\overset{c}{\sum }\theta_{c,l,a}N\left(x_{i}|\mu_{c},\Sigma_{c}\right)$$

### Statistical analysis

Parameter priors are shown in Table 1, which were assumed to be constant across pathogens. We fit the model in a Bayesian frame-work with Hamiltonian Monte Carlo No-U-Turn sampling using cmdStanR (46). Each model was fit with three chains for 3000 iterations in addition to 3000 warm-up samples. Model convergence was assessed by visual inspection of chain mixing and by R-hat convergence diagnostic across all parameters. Median and 95%CrI estimates were calculated for each parameter.

## Supplementary Material

MDAR Reproducibility Checklist

Supplementary Materials

## Figures and Tables

**Table 1:** Multivariate mixture model parameter priors. Parameter priors and limits used in model fitting.

Parameter	Parameter description	Limits	Prior
π	Prevalence	[0,1]	Beta(1, 5)
μ_0_	Mean of negative titers	[–1, inf]	Normal(0, 0.1)
μ*_R_*	Mean increase in positive titers	[0, inf]	Normal(3, 0.1)
σ_0_	SD of negative titers	[0, inf]	Normal(0.4, 0.05)
σ_1_	SD of positive titers	[0, inf]	Normal(0.7, 0.05)
ϕ	Cross-reactivity	[0, inf]	Exp(8)
ρ_0_	Correlation in negative titers	[0,1]	Beta(2, 2)
μ*_E_*	Mean of ELISA titers	[–1, inf]	Normal(2, 1)
σ*_E_*	SD of ELISA titers	[0, inf]	Normal(0.5, 0.2)

## Data Availability

All data associated with this study are present in the paper or the Supplementary Materials. Anonymized data and code to reproduce the analysis can be found at https://doi.org/10.5281/zenodo.17340210.
