# Supplementary material for: Epidemiological and antigenic inferences from serological cross-reactivity among arboviruses
Source: Sci Transl Med. Author manuscript; Available in PMC 2025 Dec 30. (PMC7618549; doi:10.1126/scitranslmed.ads8680)
Supplement: Supplementary Materials [file EMS211400-supplement-Supplementary_Materials.pdf]

Science Translational Medicine
AAAS

Supplementary Materials for

Epidemiological and antigenic inferences from serological cross-reactivity among arboviruses

Megan O'Driscoll *et al.*

Corresponding author: Megan O'Driscoll, megan.odriscoll@unige.ch; Henrik Salje, hs743@cam.ac.uk

Sci. Transl. Med. **17**, eads8680 (2025)
DOI: 10.1126/scitranslmed.ads8680

The PDF file includes:

Supplementary Materials and Methods
Figs. S1 to S9
Tables S1 to S6
References (*47–49*)

Other Supplementary Material for this manuscript includes the following:

MDAR Reproducibility Checklist

Supplementary Materials and Methods

1D mixture model & titer cutoffs

To compare the results of the multivariate Gaussian mixture model framework to traditional approaches, we fit classic single dimension (1D) Gaussian mixture models independently to the antibody titer data from each pathogen. Here, each model fits a two-component Gaussian mixture distribution, with a negative and positive component. The model likelihood is given in equation 1. The same parameter priors as the multivariate models were used for π, μ_0, μ_1, σ_0 and σ_1, whereas ϕ and ρ parameters are not estimated. Cutoffs to classify individuals as positive or negative to each pathogen were calculated as the mean of means of the two Gaussian distributions.

$$LnL = \sum_{i=1}^{n} ln((1 - \pi)N(x_i|\mu_0, \sigma_0) + \pi N(x_i|\mu_1, \sigma_1)) \quad (1)$$

Catalytic model

For pathogens where age-specific prevalence estimates increased with age indicating endemic transmission dynamics, we fit catalytic models to quantify the annual force of infection (FOI). The FOI, λ is the rate at which susceptible individuals become infected each year. We assumed that λ is constant over time and age, giving long-term averages of past FOI. The expected proportion of the population positive by age, $\hat{\pi}_a$, can then be calculated as shown in equation 2. The model was fit in a Bayesian framework using cmdStanR assuming the proportion of positive individuals in each age group to follow a binomial distribution with probability of success equal to $\hat{\pi}_a$. We used a uniform prior between 0 and 1 for λ and fit the model with 3 chains of 10,000 warmup iterations plus 10,000 sampling iterations each.

$$\hat{\pi}_a = 1 - exp^{-\lambda a} \quad (2)$$

Multidimensional scaling of antigen-sera relationships

To summarize antibody cross-reactivity estimates, we used multidimensional scaling (MDS) to translate the model median estimates to a 2D map depicting relative antibody-antigen relationships. We used only μ estimates from Gaussian components characterizing individuals infected with a single pathogen only, therefore excluding μ estimates from infection statuses that were positive to multiple pathogens or no pathogens. We used the Racmacs R package [47] for multidimensional scaling of the model Gaussian μ estimates. Scaling the antibody-antigen data to 2 dimensions, we performed 10,000 optimization runs to find the best arrangement of antigens and sera to represent their relative similarities. For each optimization run, points were randomly distributed in 2D space and a limited-memory Broyden–Fletcher–Goldfarb–Shanno (L-BFGS) gradient-based optimization algorithm is applied to find the optimal positions of the points. Antigen-sera distances were extracted from the model fit with the highest log-likelihood.

Maximum likelihood phylogeny

We reconstructed a distance-based maximum-likelihood phylogeny of the envelope domain III (EDIII) proteins from each flavivirus considered in this study. We used EDIII protein sequences from reference genomes in NCBI, with accession numbers as follows: WNV (YP 001527880.1), DENV1 (NP 722460.2), DENV2 (NP 739583.2), DENV3 (YP 001531168.2), DENV4 (NP 740317.1), JEV (NP 775666.1), TBEV (NP 775503.1), ZIKV (YP 009227198.1), YFV (NP 740305). As CHIKV is an alphavirus, we did not consider it in this analysis. We aligned all protein sequences using MUSCLE [48] and reconstructed the phylogeny using IQ-tree [49], with an LG+G4 substitution model that was automatically selected.

Simulation Study

The methods and results of the simulation study can be found on GitHub at https://github.com/meganodris/MultiSero.

Supplementary Figures

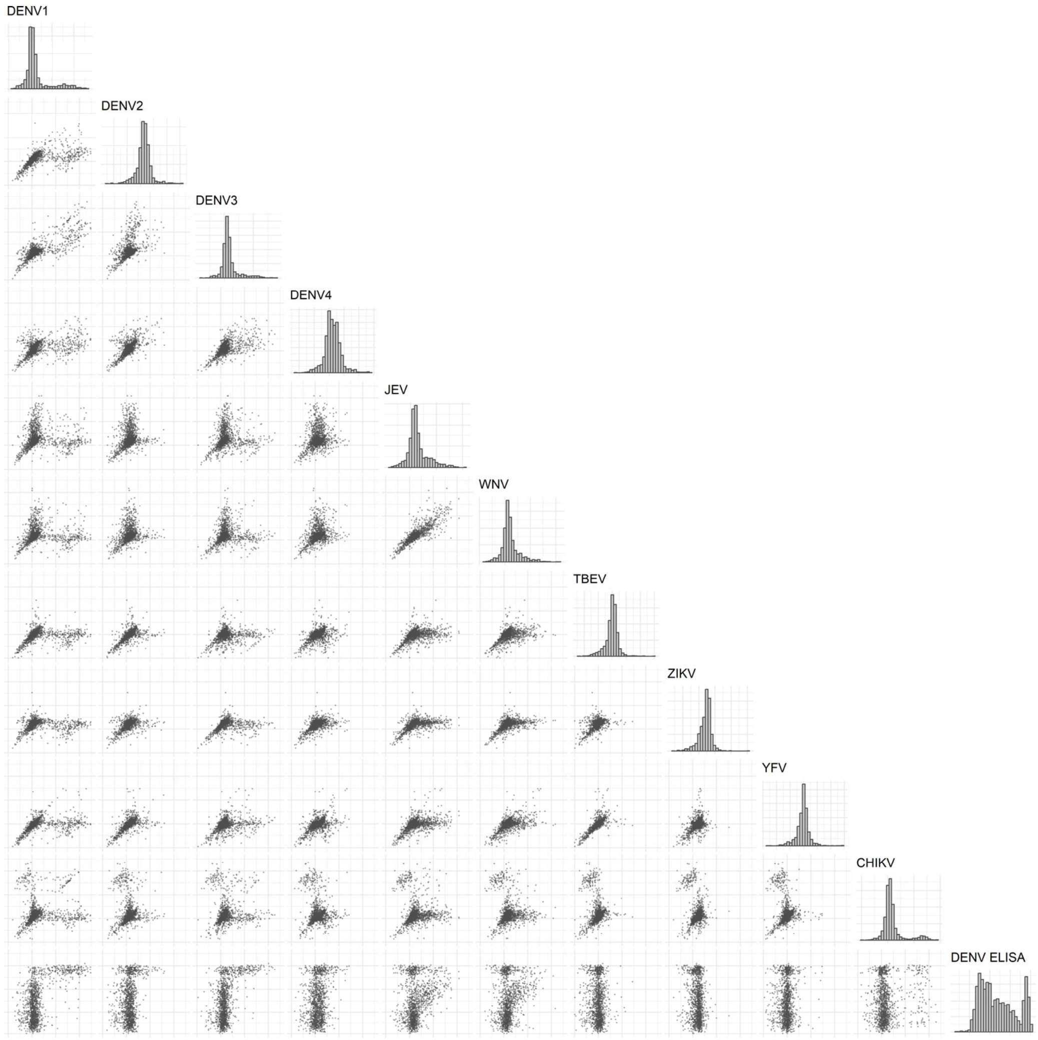

Figure S1. Antibody titer distributions across antigen pairs. Gray bars show the population distribution of measured antibody titers against each antigen, shown on a log relative fluorescence intensity (RFI) scale for the multiplex assay antigens and a log Panbio unit scale for the DENV enzyme-linked immunosorbent assay (ELISA) assay. Black points in the scatter plots show antibody titer values for each pair of antigens, where the x-axes correspond to antibodies measured against the antigen at the top of each column panels and the y-axes correspond to antibodies measured against the antigen at the right of each row panel.

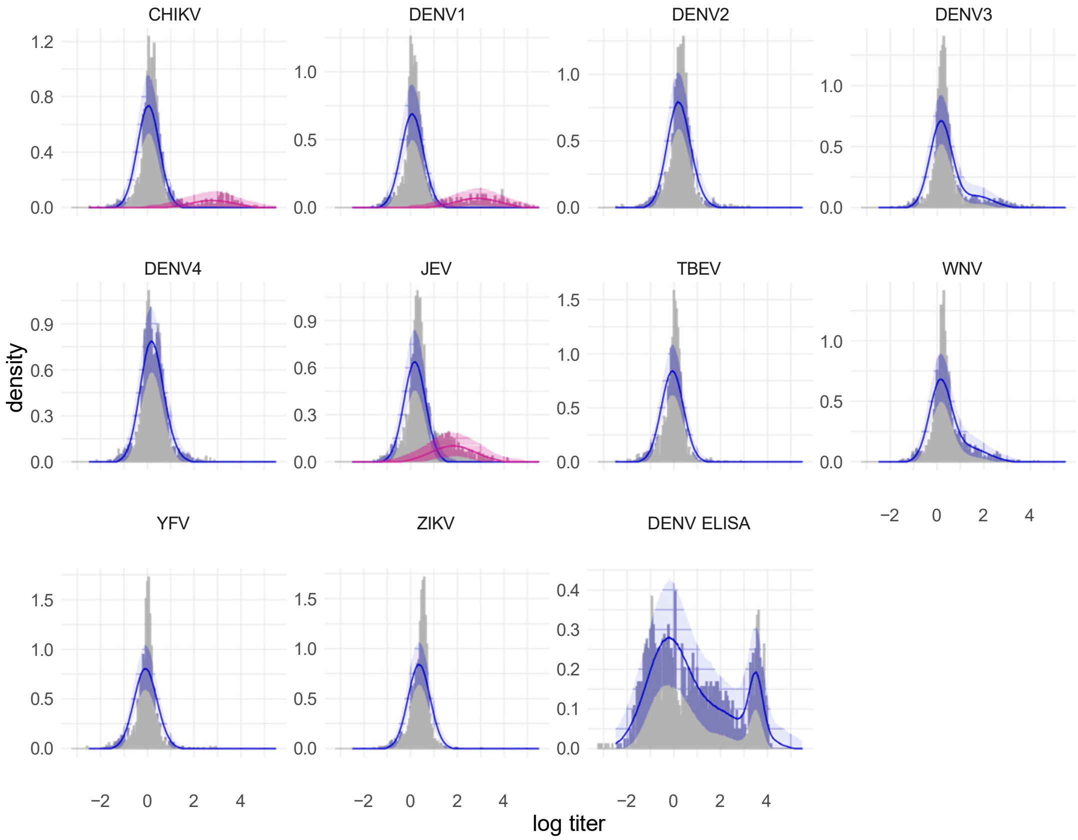

Figure S2. Model fit to multi-pathogen titer distributions. Gray bars show measured log antibody titer distributions for each pathogen. Blue lines and ribbons show median and 95% credible interval estimates of log titer distributions reconstructed by the model, encompassing negative and cross-reactive multivariate Gaussian components. Pink lines and ribbons show median and 95% credible interval estimates of log titer distributions reconstructed by the model for positive Gaussian components. Here, the DENV ELISA titers were explained by Gaussian components fit to the EDIII multiplex antigen data.

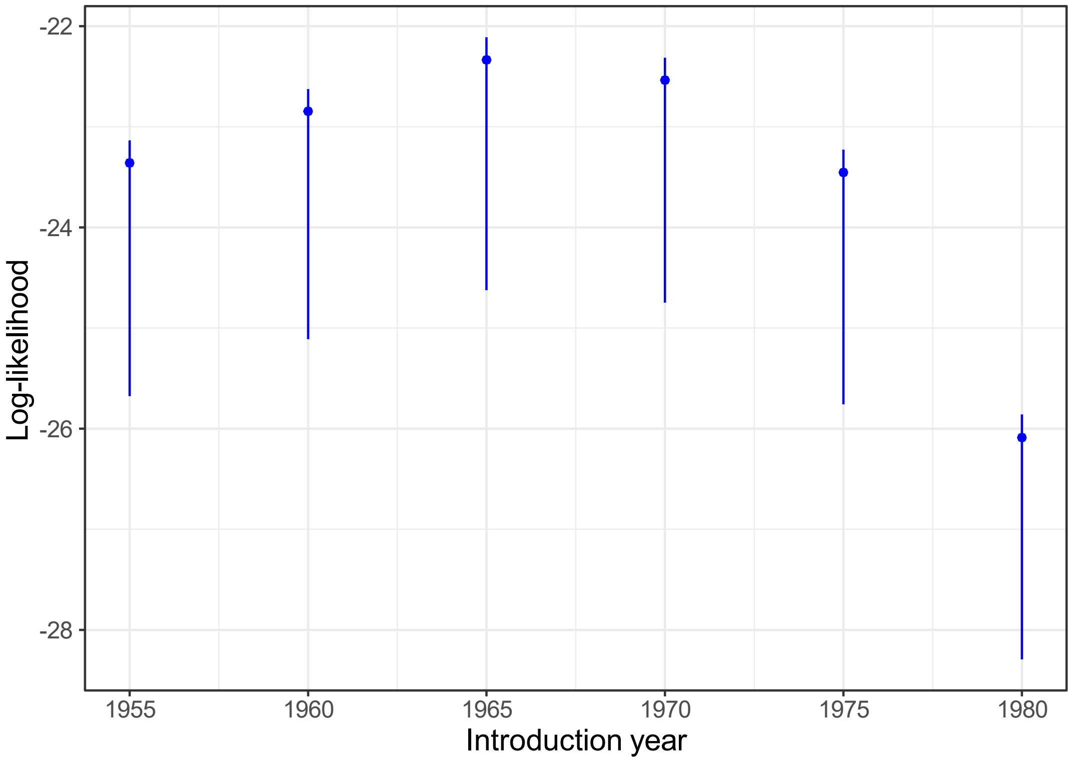

Figure S3. Catalytic model log-likelihood by assumed JEV introduction time. Blue points and lines show the model median and 95% credible interval (CrI) log-likelihood estimates by assumed introduction time of JEV.

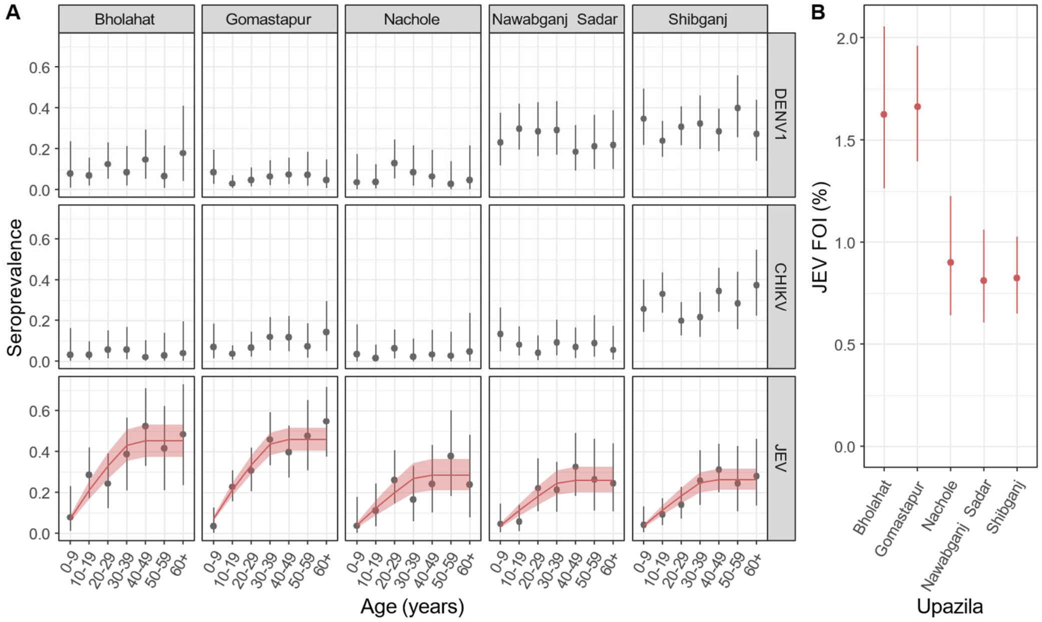

Figure S4. Infection prevalence estimates by pathogen, location, and age. (A) Shown are estimates of infection prevalence by pathogen, upazila and age group, where gray points and lines indicate median and 95%CrI estimates. Red lines and shaded ribbons show the fit of catalytic models to the infection prevalence estimates, assuming constant endemic JEV transmission since 1977. **(B)** Shown are the estimates of JEV FOI by upazila.

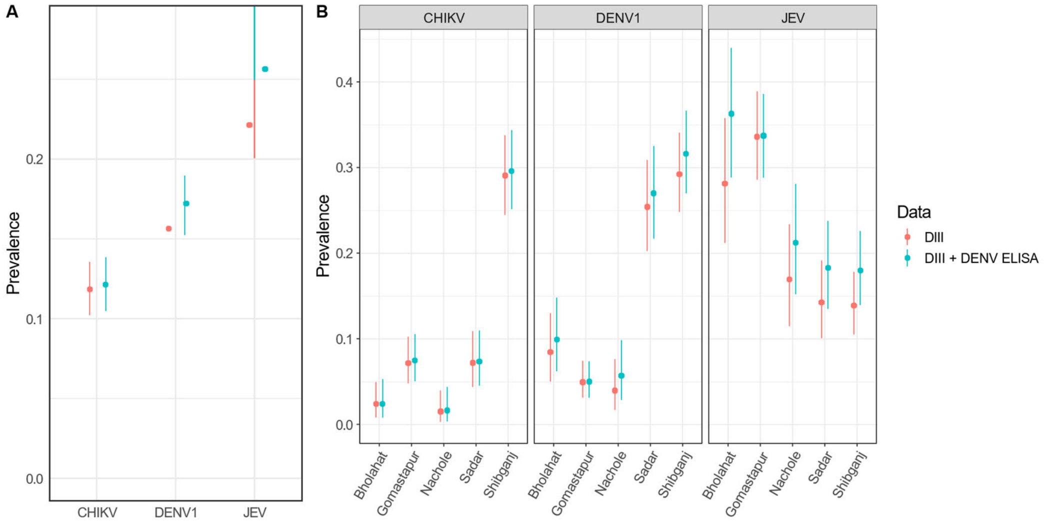

Figure S5. Infection prevalence estimates by assay data. (A) Shown are the prevalence of infection per pathogen estimated by the location and age model by assay data for the district of Chapai Nawabganj. **(B)** Shown are the prevalence of past infection estimates by pathogen and sub-district depending on which data was included in the model. Points and lines show the median and 95%CrI model estimates.

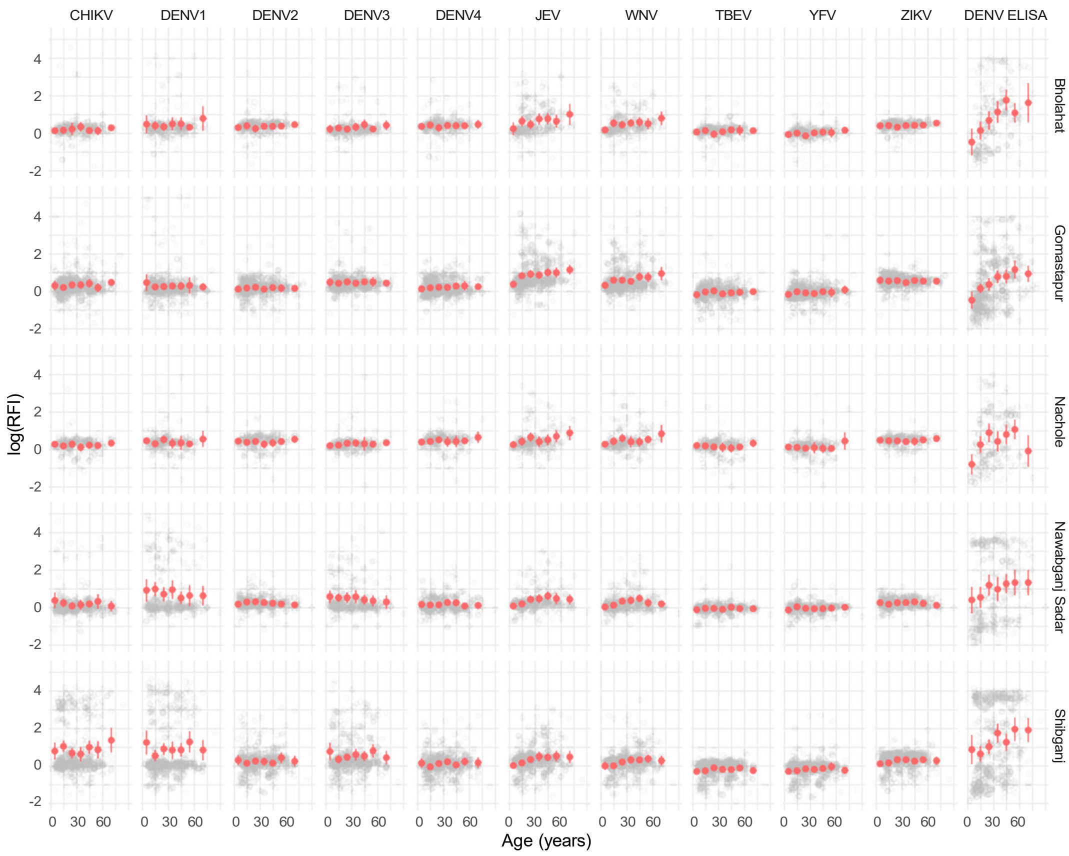

Figure S6. RFI by pathogen, location and age. Gray points show the log relative fluorescence intensity (RFI) values for each individual by age shown on the x- axis, antigen (column panels), and sub-district (row panels). Red points and lines show the population mean and 95% confidence interval estimates of log RFI by age group. Individuals were grouped by 10-year age groups from 0-60 years and individuals aged 60+ were aggregated to a single age group. Red points and lines are plotted at the respective age group mid-points.

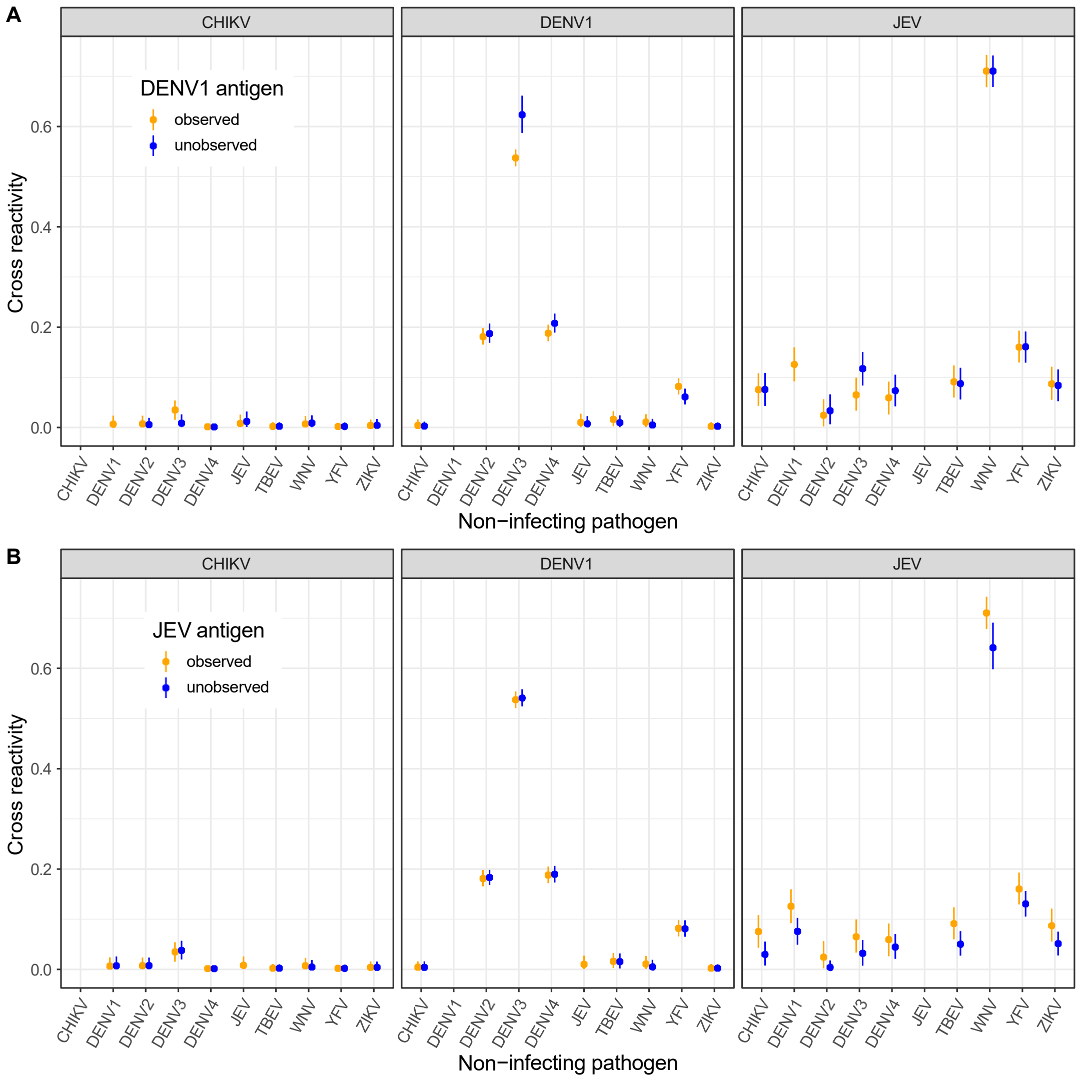

Figure S7. Reconstructed cross reactivity estimates when pathogens are unobserved. (A and B) Cross reactivity estimates, defined as relative titer increases to the non-infecting pathogen compared to the infecting pathogen, are shown for models where antigens are observed vs unobserved. Points and lines indicate median and 95%CrI model estimates. Model results are shown for analysis when the DENV1 antigen is removed (A) and when the JEV antigen is removed (B). Each sub-panel represents the infecting pathogen.

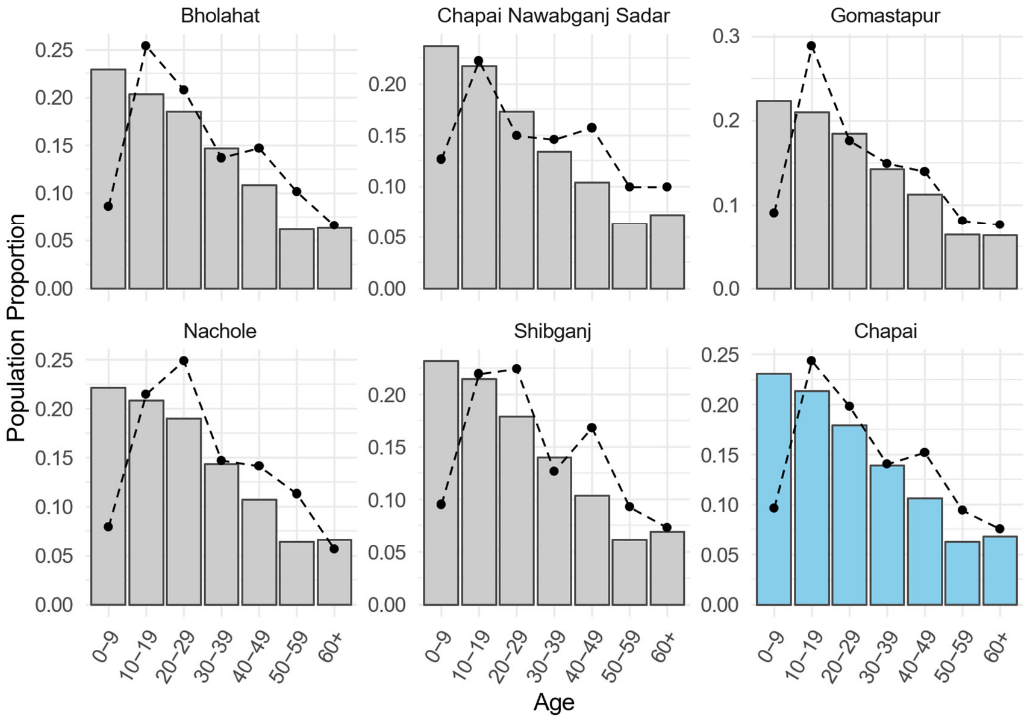

Figure S8. Age distribution of study participants. The gray bars in each panel show the population age distribution for each upazila and the blue bars show the population age distribution for the wider zila of Chapai Nawabganj (Chapai). Black points and dashed lines indicate the proportion of study participants in each age group by upazila and across the total study population representing Chapai.

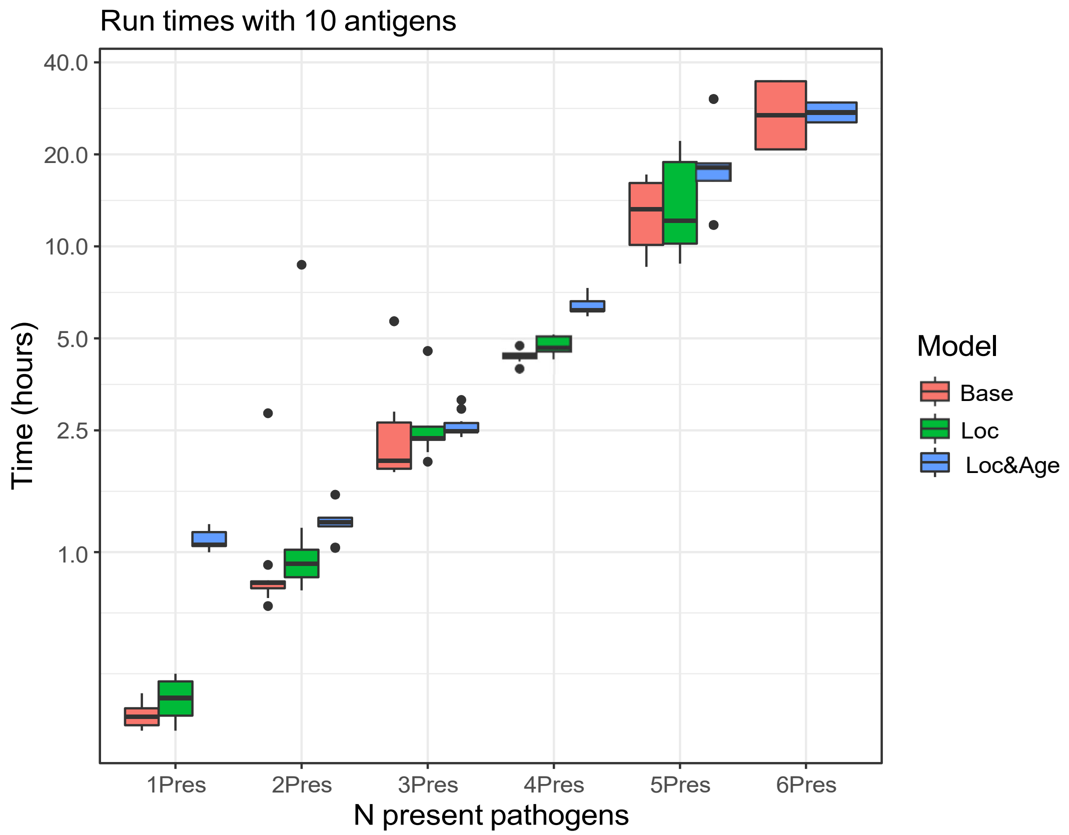

Figure S9. Model run times by number of present pathogens. Model run times are shown for each model variant - base model, location-specific model (Loc) and the age- and location-specific model (Loc&Age). Models were fit to 10 multiplex antigens plus the DENV ELISA assay. Each model was run with 3 parallel chains of 3,000 warm-up plus 3,000 sampling iterations each on a high performance computing cluster.

Supplementary Tables

Table S1. Variable selection process model metrics for base model. Log-likelihood, likelihood increment percentage (LIP) and LIP per component (LIPpc) values from the base model. N components is the number of multivariate Gaussian components used to characterize the data for each model.

N Present Pathogens	N Components	Present Pathogen	LogLik	LIP	LIPpc
1	2	CHIKV	-16,163.1	-	-
		DENV1	-14,943.2	-	-
		DENV2	-16.086.9	-	-
		DENV3	-15,319.9	-	-
		DENV4	-16,097.9	-	-
		JEV	-16,741.4	-	-
		TBEV	-16,256.8	-	-
		WNV	-16,716.1	-	-
		YFV	-16,217.7	-	-
		ZIKV	-16,778.2	-	-
2	4	CHIKV	-12,281.0	17.8%	8.9%
		DENV2	-14,853.0	0.6%	0.3%
		DENV3	-14,491.1	3.0%	1.5%
		DENV4	-14,215.5	4.9%	2.4%
		JEV	-13,726.0	8.1%	4.1%
		TBEV	-14,015.4	6.2%	3.1%
		WNV	-13,764.6	7.9%	3.9%
		YFV	-14,046.7	6.0%	3.0%
		ZIKV	-14,916.0	0.2%	0.1%
3	8	DENV2	-12,118.3	1.3%	0.3%
		DENV3	-11,944.6	2.7%	0.7%
		DENV4	-11,753.4	4.3%	1.1%
		JEV	-9,847.1	19.8%	5.0%
		TBEV	-11,474.8	6.6%	1.6%
		WNV	-10,170.7	17.2%	4.3%
		YFV	-11,324.8	7.8%	1.9%
		ZIKV	-12,268.5	0.1%	0.0%
4	16	DENV2	-9,545.2	3.1%	0.4%
		DENV3	-9,210.1	6.5%	0.8%
		DENV4	-9,450.7	4.0%	0.5%
		TBEV	-9,763.2	0.9%	0.1%
		WNV	-9,621.4	2.3%	0.3%
		YFV	-9,673.8	1.8%	0.2%
		ZIKV	-9,642.5	2.1%	0.3%

Table S2. Variable selection process model metrics for location-specific model. Log-likelihood, LIP and LIPpc values from the location-specific model. N components is the number of multivariate Gaussian components used to characterize the data for each model.

N Present Pathogens	N Components	Present Pathogen	Log-Likelihood	LIP	LIPpc
1	2	CHIKV	-16,082.6	-	-
		DENV1	-14,877.4	-	-
		DENV2	-16,038.5	-	-
		DENV3	-15,253.8	-	-
		DENV4	-16,034.2	-	-
		JEV	-16,733.1	-	-
		TBEV	-16,787.8	-	-
		WNV	-16,705.8	-	-
		YFV	-16,163.1	-	-
		ZIKV	-16,722.5	-	-
2	4	CHIKV	-12,136.0	18.4%	9.2%
		DENV2	-14,798.2	0.5%	0.3%
		DENV3	-14,414.6	3.1%	1.6%
		DENV4	-14,076.6	5.4%	2.7%
		JEV	-13,653.7	8.2%	4.1%
		TBEV	-13,883.3	6.7%	3.3%
		WNV	-13,694.7	7.9%	4.0%
		YFV	-13,905.3	6.5%	3.3%
		ZIKV	-14,846.6	0.2%	0.1%
3	8	DENV2	-11,968.0	1.4%	0.3%
		DENV3	-11,793.9	2.8%	0.7%
		DENV4	-12,023.7	0.9%	0.2%
		JEV	-9,690.3	20.2%	5.0%
		TBEV	-11,328.5	6.6%	1.7%
		WNV	-10,015.9	17.5%	4.4%
		YFV	-11,171.5	7.9%	2.0%
		ZIKV	-12,123.9	0.1%	0.0%
4	16	DENV2	-9,379.6	3.2%	0.4%
		DENV3	-9,129.2	5.8%	0.7%
		DENV4	-9,278.0	4.3%	0.5%
		TBEV	-9,605.1	0.9%	0.1%
		WNV	-9,461.0	2.4%	0.3%
		YFV	-9,517.2	1.8%	0.2%
		ZIKV	-9,477.3	2.2%	0.3%

Table S3. Variable selection process model metrics for age- and location-specific model. Log-likelihood, LIP and LIPpc values from the age- and location-specific model. N components is the number of multivariate Gaussian components used to characterize the data for each model.

N Present Pathogens	N Components	Present Pathogen	Log-Likelihood	LIP	LIPpc
1	2	CHIKV	-16,088.5	-	-
		DENV1	-14,877.8	-	-
		DENV2	-16,037.6	-	-
		DENV3	-15,253.7	-	-
		DENV4	-16,036.2	-	-
		JEV	-16,990.6	-	-
		TBEV	-16,203.2	-	-
		WNV	-16,678.5	-	-
		YFV	-16,163.7	-	-
		ZIKV	-17,575.4	-	-
2	4	CHIKV	-12,138.7	18.4%	9.2%
		DENV2	-14,804.9	0.5%	0.2%
		DENV3	-14,422.4	3.1%	1.5%
		DENV4	-14,077.9	5.4%	2.7%
		JEV	-13,589.8	8.7%	4.3%
		TBEV	-13,882.6	6.7%	3.3%
		WNV	-13,638.6	8.3%	4.2%
		YFV	-13,908.7	6.5%	3.3%
		ZIKV	-14,863.3	0.1%	0.1%
3	8	DENV2	-11,971.8	1.4%	0.3%
		DENV3	-11,797.3	2.8%	0.7%
		DENV4	-12,031.3	0.9%	0.2%
		JEV	-9,638.0	20.6%	5.2%
		TBEV	-11,314.5	6.8%	1.7%
		WNV	-9,960.2	17.9%	4.5%
		YFV	-11,149.8	8.1%	2.0%
		ZIKV	-12,108.7	0.2%	0.1%
4	16	DENV2	-9,330.2	3.2%	0.4%
		DENV3	-9,079.4	5.8%	0.7%
		DENV4	-9,229.0	4.2%	0.5%
		TBEV	-9,565.2	0.8%	0.1%
		WNV	-9,404.0	2.4%	0.3%
		YFV	-9,471.1	1.7%	0.2%
		ZIKV	-9,430.2	2.2%	0.3%
5	32	DENV2	-8,720.5	4.0%	0.2%
		DENV4	-8,474.5	6.7%	0.4%
		TBEV	-8,653.4	4.7%	0.3%
		WNV	-8,730.9	3.8%	0.2%
		YFV	-8,880.1	2.2%	0.1%
		ZIKV	-8,773.1	3.4%	0.2%

6	64	DENV2	-8,130.7	4.1%	0.1%
		TBEV	-8,321.9	1.8%	0.1%
		WNV	-8,004.6	5.5%	0.2%
		YFV	-8,186.3	3.4%	0.1%
		ZIKV	-8,397.1	0.9%	0.0%

Table S4. Prevalence estimates from final model. Median and 95% CrI estimates of pathogen prevalence by pathogen, age group, and upazila (subdistrict).

Pathogen	Location	Age Group	Prevalence (95% CrI)
DENV1	Chapai Nawabganj Sadar Upazila	0-4	0.08 (0.00–0.39)
DENV1	Chapai Nawabganj Sadar Upazila	5-9	0.25 (0.13–0.41)
DENV1	Chapai Nawabganj Sadar Upazila	10-19	0.30 (0.20–0.42)
DENV1	Chapai Nawabganj Sadar Upazila	20-29	0.29 (0.16–0.43)
DENV1	Chapai Nawabganj Sadar Upazila	30-39	0.29 (0.17–0.43)
DENV1	Chapai Nawabganj Sadar Upazila	40-49	0.19 (0.09–0.32)
DENV1	Chapai Nawabganj Sadar Upazila	50-59	0.21 (0.10–0.38)
DENV1	Chapai Nawabganj Sadar Upazila	60+	0.22 (0.10–0.39)
DENV1	Shibganj Upazila	0-4	0.08 (0.00–0.37)
DENV1	Shibganj Upazila	5-9	0.37 (0.23–0.53)
DENV1	Shibganj Upazila	10-19	0.24 (0.16–0.33)
DENV1	Shibganj Upazila	20-29	0.31 (0.22–0.41)
DENV1	Shibganj Upazila	30-39	0.32 (0.20–0.46)
DENV1	Shibganj Upazila	40-49	0.29 (0.19–0.40)
DENV1	Shibganj Upazila	50-59	0.40 (0.26–0.56)
DENV1	Shibganj Upazila	60+	0.27 (0.14–0.45)
DENV1	Gomastapur Upazila	0-4	0.08 (0.00–0.37)
DENV1	Gomastapur Upazila	5-9	0.09 (0.03–0.21)
DENV1	Gomastapur Upazila	10-19	0.03 (0.01–0.07)
DENV1	Gomastapur Upazila	20-29	0.05 (0.01–0.11)
DENV1	Gomastapur Upazila	30-39	0.07 (0.02–0.14)
DENV1	Gomastapur Upazila	40-49	0.08 (0.03–0.16)
DENV1	Gomastapur Upazila	50-59	0.07 (0.02–0.19)
DENV1	Gomastapur Upazila	60+	0.05 (0.01–0.15)
DENV1	Nachole Upazila	0-4	0.07 (0.00–0.34)
DENV1	Nachole Upazila	5-9	0.05 (0.00–0.21)
DENV1	Nachole Upazila	10-19	0.04 (0.01–0.12)
DENV1	Nachole Upazila	20-29	0.13 (0.05–0.24)
DENV1	Nachole Upazila	30-39	0.09 (0.02–0.21)
DENV1	Nachole Upazila	40-49	0.06 (0.01–0.19)
DENV1	Nachole Upazila	50-59	0.03 (0.00–0.14)
DENV1	Nachole Upazila	60+	0.05 (0.00–0.22)
DENV1	Bholahat Upazila	0-4	0.06 (0.00–0.31)
DENV1	Bholahat Upazila	5-9	0.10 (0.02–0.31)
DENV1	Bholahat Upazila	10-19	0.07 (0.02–0.16)
DENV1	Bholahat Upazila	20-29	0.12 (0.05–0.24)
DENV1	Bholahat Upazila	30-39	0.08 (0.02–0.21)
DENV1	Bholahat Upazila	40-49	0.15 (0.05–0.29)
DENV1	Bholahat Upazila	50-59	0.07 (0.01–0.21)

DENV1	Bholahat Upazila	60+	0.18 (0.05–0.41)
CHIKV	Chapai Nawabganj Sadar Upazila	0-4	0.08 (0.00–0.38)
CHIKV	Chapai Nawabganj Sadar Upazila	5-9	0.15 (0.05–0.29)
CHIKV	Chapai Nawabganj Sadar Upazila	10-19	0.08 (0.03–0.17)
CHIKV	Chapai Nawabganj Sadar Upazila	20-29	0.04 (0.01–0.13)
CHIKV	Chapai Nawabganj Sadar Upazila	30-39	0.09 (0.03–0.20)
CHIKV	Chapai Nawabganj Sadar Upazila	40-49	0.07 (0.02–0.17)
CHIKV	Chapai Nawabganj Sadar Upazila	50-59	0.09 (0.02–0.22)
CHIKV	Chapai Nawabganj Sadar Upazila	60+	0.06 (0.01–0.17)
CHIKV	Shibganj Upazila	0-4	0.09 (0.00–0.37)
CHIKV	Shibganj Upazila	5-9	0.28 (0.15–0.43)
CHIKV	Shibganj Upazila	10-19	0.33 (0.24–0.43)
CHIKV	Shibganj Upazila	20-29	0.20 (0.13–0.29)
CHIKV	Shibganj Upazila	30-39	0.21 (0.11–0.35)
CHIKV	Shibganj Upazila	40-49	0.35 (0.24–0.47)
CHIKV	Shibganj Upazila	50-59	0.29 (0.15–0.44)
CHIKV	Shibganj Upazila	60+	0.37 (0.23–0.55)
CHIKV	Gomastapur Upazila	0-4	0.19 (0.02–0.52)
CHIKV	Gomastapur Upazila	5-9	0.05 (0.01–0.16)
CHIKV	Gomastapur Upazila	10-19	0.04 (0.01–0.08)
CHIKV	Gomastapur Upazila	20-29	0.07 (0.02–0.15)
CHIKV	Gomastapur Upazila	30-39	0.12 (0.05–0.22)
CHIKV	Gomastapur Upazila	40-49	0.12 (0.05–0.23)
CHIKV	Gomastapur Upazila	50-59	0.07 (0.02–0.18)
CHIKV	Gomastapur Upazila	60+	0.14 (0.05–0.29)
CHIKV	Nachole Upazila	0-4	0.08 (0.00–0.33)
CHIKV	Nachole Upazila	5-9	0.05 (0.00–0.22)
CHIKV	Nachole Upazila	10-19	0.02 (0.00–0.08)
CHIKV	Nachole Upazila	20-29	0.06 (0.02–0.16)
CHIKV	Nachole Upazila	30-39	0.02 (0.00–0.11)
CHIKV	Nachole Upazila	40-49	0.03 (0.00–0.15)
CHIKV	Nachole Upazila	50-59	0.03 (0.00–0.14)
CHIKV	Nachole Upazila	60+	0.05 (0.00–0.23)
CHIKV	Bholahat Upazila	0-4	0.07 (0.00–0.31)
CHIKV	Bholahat Upazila	5-9	0.04 (0.00–0.21)
CHIKV	Bholahat Upazila	10-19	0.03 (0.00–0.10)
CHIKV	Bholahat Upazila	20-29	0.06 (0.01–0.15)
CHIKV	Bholahat Upazila	30-39	0.06 (0.01–0.17)
CHIKV	Bholahat Upazila	40-49	0.02 (0.00–0.11)
CHIKV	Bholahat Upazila	50-59	0.03 (0.00–0.13)
CHIKV	Bholahat Upazila	60+	0.04 (0.00–0.19)
JEV	Chapai Nawabganj Sadar Upazila	0-4	0.09 (0.00–0.38)

JEV	Chapai Nawabganj Sadar Upazila	5-9	0.05 (0.01–0.16)
JEV	Chapai Nawabganj Sadar Upazila	10-19	0.06 (0.01–0.14)
JEV	Chapai Nawabganj Sadar Upazila	20-29	0.22 (0.11–0.37)
JEV	Chapai Nawabganj Sadar Upazila	30-39	0.21 (0.11–0.36)
JEV	Chapai Nawabganj Sadar Upazila	40-49	0.33 (0.19–0.48)
JEV	Chapai Nawabganj Sadar Upazila	50-59	0.26 (0.11–0.46)
JEV	Chapai Nawabganj Sadar Upazila	60+	0.24 (0.10–0.43)
JEV	Shibganj Upazila	0-4	0.09 (0.00–0.38)
JEV	Shibganj Upazila	5-9	0.04 (0.01–0.14)
JEV	Shibganj Upazila	10-19	0.09 (0.04–0.17)
JEV	Shibganj Upazila	20-29	0.14 (0.07–0.23)
JEV	Shibganj Upazila	30-39	0.26 (0.14–0.40)
JEV	Shibganj Upazila	40-49	0.31 (0.20–0.43)
JEV	Shibganj Upazila	50-59	0.25 (0.12–0.42)
JEV	Shibganj Upazila	60+	0.28 (0.13–0.46)
JEV	Gomastapur Upazila	0-4	0.08 (0.00–0.37)
JEV	Gomastapur Upazila	5-9	0.04 (0.00–0.14)
JEV	Gomastapur Upazila	10-19	0.23 (0.16–0.31)
JEV	Gomastapur Upazila	20-29	0.31 (0.21–0.42)
JEV	Gomastapur Upazila	30-39	0.46 (0.34–0.59)
JEV	Gomastapur Upazila	40-49	0.39 (0.27–0.53)
JEV	Gomastapur Upazila	50-59	0.48 (0.31–0.66)
JEV	Gomastapur Upazila	60+	0.55 (0.37–0.72)
JEV	Nachole Upazila	0-4	0.07 (0.00–0.33)
JEV	Nachole Upazila	5-9	0.05 (0.00–0.22)
JEV	Nachole Upazila	10-19	0.11 (0.03–0.24)
JEV	Nachole Upazila	20-29	0.26 (0.15–0.41)
JEV	Nachole Upazila	30-39	0.17 (0.06–0.33)
JEV	Nachole Upazila	40-49	0.24 (0.10–0.43)
JEV	Nachole Upazila	50-59	0.38 (0.19–0.60)
JEV	Nachole Upazila	60+	0.24 (0.08–0.49)
JEV	Bholahat Upazila	0-4	0.07 (0.00–0.31)
JEV	Bholahat Upazila	5-9	0.10 (0.02–0.30)
JEV	Bholahat Upazila	10-19	0.29 (0.17–0.42)
JEV	Bholahat Upazila	20-29	0.24 (0.13–0.40)
JEV	Bholahat Upazila	30-39	0.39 (0.22–0.58)
JEV	Bholahat Upazila	40-49	0.52 (0.33–0.71)
JEV	Bholahat Upazila	50-59	0.42 (0.22–0.63)
JEV	Bholahat Upazila	60+	0.49 (0.24–0.74)

Table S5. Cross-reactivity estimates from final model. Median and 95% CrI estimates of between-pathogen cross-reactivity, defined as the relative titer increase in the measured pathogen compared to the infecting pathogen.

Infecting Pathogen	Measured Pathogen	Cross-Reactivity (95% CrI)
DENV1	CHIKV	0.00 (0.00–0.02)
DENV1	JEV	0.01 (0.00–0.03)
DENV1	DENV2	0.18 (0.16–0.20)
DENV1	DENV3	0.54 (0.52–0.55)
DENV1	DENV4	0.19 (0.17–0.20)
DENV1	WNV	0.01 (0.00–0.03)
DENV1	TBEV	0.02 (0.00–0.03)
DENV1	YFV	0.08 (0.07–0.10)
DENV1	ZIKV	0.00 (0.00–0.01)
CHIKV	DENV1	0.01 (0.00–0.02)
CHIKV	JEV	0.01 (0.00–0.03)
CHIKV	DENV2	0.01 (0.00–0.02)
CHIKV	DENV3	0.03 (0.02–0.05)
CHIKV	DENV4	0.00 (0.00–0.01)
CHIKV	WNV	0.01 (0.00–0.02)
CHIKV	TBEV	0.00 (0.00–0.01)
CHIKV	YFV	0.00 (0.00–0.01)
CHIKV	ZIKV	0.00 (0.00–0.02)
JEV	DENV1	0.13 (0.09–0.16)
JEV	CHIKV	0.08 (0.04–0.11)
JEV	DENV2	0.03 (0.00–0.06)
JEV	DENV3	0.07 (0.03–0.10)
JEV	DENV4	0.06 (0.03–0.09)
JEV	WNV	0.71 (0.68–0.74)
JEV	TBEV	0.09 (0.06–0.13)
JEV	YFV	0.16 (0.13–0.19)
JEV	ZIKV	0.09 (0.06–0.12)

Table S6. Gaussian component parameter estimates from final model. Median and 95% CrI estimates of Gaussian component parameters.

Parameter	Pathogen	Median (95% CrI)
μ_0	DENV1	0.01 (-0.01-0.04)
	DENV2	0.13 (0.11-0.16)
	DENV3	0.13 (0.10-0.16)
	DENV4	0.10 (0.07-0.13)
	CHIKV	0.01 (-0.01-0.04)
	JEV	0.17 (0.15-0.20)
	WNV	0.13 (0.11-0.16)
	TBEV	-0.11 (-0.14- -0.08)
	YFV	-0.18 (-0.20- -0.15)
	ZIKV	0.32 (0.30-0.35)
	DENV ELISA	-0.30 (-0.37- -0.22)
μ_R	DENV1	2.87 (2.76-2.97)
	CHIKV	2.79 (2.65-2.92)
	JEV	1.82 (1.72-1.93)
σ_0	All	0.47 (0.46-0.48)
σ_1	All	0.96 (0.91-1.01)
ρ_{00}	All	0.56 (0.54-0.58)
